# Supraparticles from Cubic Iron Oxide Nanoparticles: Synthesis, Polymer Encapsulation, Functionalization, and Magnetic Properties

**DOI:** 10.1021/acs.langmuir.4c02753

**Published:** 2024-10-18

**Authors:** Lea R. Klauke, Michael Kampferbeck, Malte Holzapfel, Neus Feliu, Benedikt Sochor, Sarathlal Koyiloth Vayalil, Andreas Meyer, Tobias Vossmeyer

**Affiliations:** † Institute of Physical Chemistry, University of Hamburg, Grindelallee 117, 20146 Hamburg, Germany; ‡ Center for Applied Nanotechnology (CAN), Fraunhofer Institute for Applied Polymer Research (IAP), Grindelallee 117, 20146 Hamburg, Germany; § Deutsches Elektron Synchrotron (DESY), Notkestraße 85, 20607 Hamburg, Germany; ∥ Applied Science Cluster, University of Petroleum and Energy Studies (UPES), Dehradun 248007, India

## Abstract

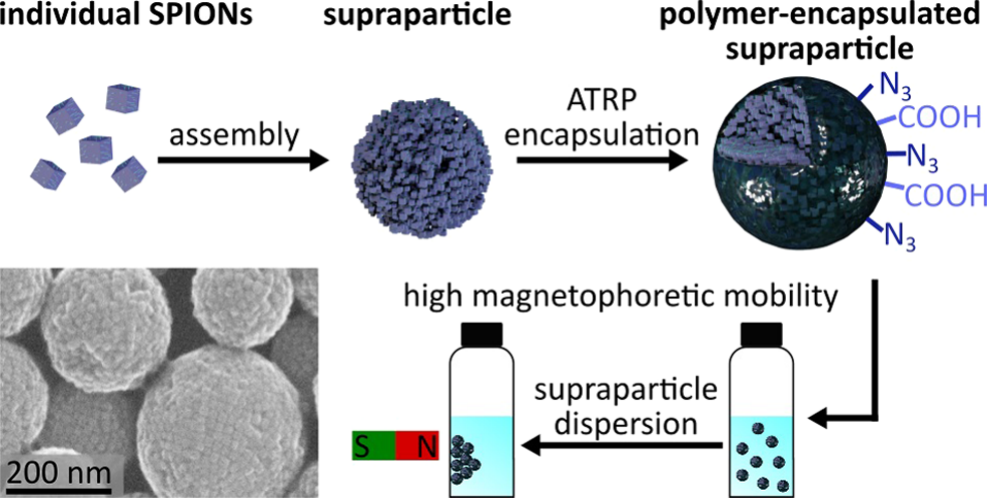

Supraparticles (SPs) consisting of superparamagnetic iron oxide nanoparticles (SPIONs) are of great interest for biomedical applications and magnetic separation. To enable their functionalization with biomolecules and to improve their stability in aqueous dispersion, polymer shells are grown on the SPs’ surface. Robust polymer encapsulation and functionalization is achieved via atom transfer radical polymerization (ATRP), improving the reaction control compared to free radical polymerizations. This study presents the emulsion-based assembly of differently sized cubic SPIONs (12–30 nm) into SPs with diameters ranging from ∼200 to ∼400 nm using dodecyltrimethylammonium bromide (DTAB) as the surfactant. The successful formation of well-defined spherical SPs depends upon the method used for mixing the SPION dispersion with the surfactant solution and requires the precise adjustment of the surfactant concentration. After purification, the SPs are encapsulated by growing surface-grafted polystyrene shells via activators generated by electron transfer (AGET) ATRP. The polymer shell can be decorated with functional groups (azide and carboxylate) using monomer blends for the polymerization reaction. When the amount of the monomer is varied, the shell thickness as well as the interparticle distances between the encapsulated SPIONs can be tuned with nanometer-scale precision. Small-angle X-ray scattering (SAXS) reveals that cubic SPIONs form less ordered assemblies within the SPs than spherical SPIONs. As shown by vibrating sample magnetometer measurements, the encapsulated SPs feature the same superparamagnetic behavior as their SPION building blocks. The saturation magnetization ranges between 10 and 30 emu/g and depends upon the nanocubes’ size and phase composition.

## Introduction

The fabrication of polymer beads loaded with magnetic materials is of great interest for biomedical applications, like drug delivery or magnetic particle imaging.^1^ Especially, superparamagnetic iron oxide nanoparticles (SPIONs) are being investigated as magnetic materials due to their low toxicity and biocompatibility.^1^ In general, ferrimagnetic iron oxide nanoparticles only show superparamagnetic behavior if their diameter is below 30 nm.^2^ To preserve the superparamagnetic properties of iron oxide nanoparticles and simultaneously increase the size to diameters above 30 nm, nanoparticles can be assembled into three-dimensional supraparticles (SPs). In such SPs, the magnetophoretic mobility is increased, compared to individual SPIONs.^3^ Therefore, they can be used for in vitro applications as magnetic carriers^4^ for the purification and separation of, e.g., biomolecules from complex mixtures or in magnetic lateral flow immunoassays.^5^

The encapsulation of individual nanoparticles or SPs within polymer shells can be conducted via different techniques.^6−9^ For example, presynthesized polyisoprene polymers modified with functional groups were used to exchange the initial ligands on the nanoparticle’s surface. The modified nanoparticles were then transferred into water with diblock copolymers to form micellar structures that stabilize the nanoparticles in the aqueous phase. Afterward, the surface ligands and polymer shell can be cross-linked via a thermally initiated radical polymerization.^10^ As an alternative approach, hydrophobic nanoparticles can be transferred to the aqueous medium using commercially available nonionic surfactants. The formed nanoparticle-loaded micelles can then be used as seeds in a seeded emulsion free radical polymerization (FRP) resulting in the encapsulation of nanoparticles within a polymer matrix.^11^ For example, Paquet et al.^12^ encapsulated iron oxide SPs via FRP. However, in FRPs, the reaction control is limited due to fast polymerization rates.^13^ In contrast, controlled radical polymerization (CRP) techniques improve reaction control by suppression of undesired side reactions, enabling the synthesis of polymers with defined composition and topology.^14,15^ Basically, there are three types of CRPs that can be conducted in homogeneous systems: First, nitroxide-mediated living radical polymerization^16^ (NMP), which usually requires higher reaction temperatures.^17^ Second, reversible addition–fragmentation chain transfer^18^ (RAFT) polymerization, which has been used for the synthesis of magnetic polymer beads by Bourgeat-Lami and co-workers.^19^ To graft the polymer from the SP surface, they conducted a surface modification using a presynthesized MakroRAFT initiator. Third, atom transfer radical polymerization^14,20^ (ATRP), which was used in our present study. ATRP enables a higher tolerance to basic synthesis conditions^15^ and has already been used for the encapsulation of individual SPIONs in our group.^21^

In 1998, ATRP was first carried out in an emulsion system,^22^ making the reaction more environmentally friendly, enabling increased heat dissipation, and a lower viscosity of the reaction mixture.^23^ Subsequently, the need to improve the mass transfer of the hydrophobic monomers into the emulsion led to the development of ATRP in mini-^24^ or microemulsion.^21,25^ Additionally, the initiation technique was improved using activators generated by electron transfer^26,27^ (AGET), rendering storage and handling of the catalyst under ambient atmosphere possible. With this approach, more stable Cu(II) catalysts are used to substitute catalysts based on oxygen sensitive Cu(I).

Besides the improved polymerization control, surface-grafting is another advantage of ATRP. Surface grafting is based on special ligands that bind to surfaces via anchoring groups and initiate the ATRP.^28,29^ In ATRP, surface grafting has been used to modify the surface of different macroscopic materials, including glass, silicon, metals, and natural materials.^29,30^ Further, surface grafting has been used to modify nanoscopic materials, e.g., carbon nanotubes and nanoparticles.^29,30^ Most surface-grafted ATRP reactions mentioned above have in common that they utilize initiator ligands with carboxylic acid or silane anchoring groups which can detach from the surface due to weak binding, or which easily undergo undesired homocondensation reactions, respectively.^31−33^ In contrast, phosphonic acids^21,28^ or catechols^34^ can be used as anchoring groups to overcome these problems.

In a few studies, ATRP has also been used for the encapsulation of SPs and to control the interaction between colloidal particles. For example, Ohno et al.^35^ coated iron oxide nanoparticle assemblies with a silica shell to create structural colors. Subsequently, an ATRP initiator ligand with a silane anchoring group was grafted to the silica surface and an ATRP reaction was carried out to grow a polymer shell with precisely controlled thickness. The group of Kegel^36^ used colloids of chlorinated polystyrene and grafted poly(*t*-butyl acrylate) onto their surface via ATRP to adjust the hydrophobicity of resulting colloids. To this end, a Cu(I) catalyst was used. Afterward, the resulting colloids were used to study how variations of surface properties control their assembly into differently shaped clusters. So far, however, the surface grafted encapsulation and functionalization of SPs from SPIONs via AGET ATRP with direct grafting of the initiator ligand to the iron oxide surface, has not been reported.

Here, we present the synthesis of polystyrene-encapsulated SPs from cubic SPIONs via AGET ATRP. The assembly of the cubes into SPs was achieved via evaporation-induced self-assembly (EISA), a method which was previously reported by Paquet et al.^12^ and Bai et al.^37^ After the EISA process, the initial oleic acid ligands on the nanocube surfaces inside the SPs were exchanged for the ATRP initiator bearing a phosphonic acid anchoring group. Surface grafting of the initiator ligand to the nanocubes was confirmed via Fourier transform infrared (FTIR) spectroscopy. Further, electron microscopy and thermogravimetric analysis (TGA) confirmed that the thickness of formed polystyrene shells can be adjusted by varying the volume of the added monomer. FTIR confirmed the incorporation of azide and carboxylic acid groups via the copolymerization of vinylbenzyl azide or vinylbenzoic acid. In addition, the crystallinity of the nanocube SPs (in comparison to SPs from spherical SPIONs) and swelling of the SPs after AGET ATRP encapsulation were studied using small-angle X-ray scattering (SAXS). Finally, the magnetization of SPs from differently sized nanocubes was characterized using a vibrating sample magnetometer (VSM).

## Experimental Section

### Materials

All chemicals were used without further purification if not stated elsewise. Iron oxide hydroxide (α-FeOOH, goethite, 99%) was purchased from Alfa Aesar or from Sigma-Aldrich (goethite, 30–63% Fe). Divinylbenzene (DVB, 99%, isomeric mixture), potassium chloride (99.5%), and styrene (99%) were obtained from Merck. Styrene and DVB were distilled to remove the inhibitor and stored at −20 °C prior use. l-Ascorbic acid (99%), Brij S20, 11-(2-bromoisobutyrate)-undecyl-1-phosphonic acid (BiB-UDPA, 95%), copper(II)bromide (99%), dodecyltrimethylammonium bromide (DTAB, 98%), 1-octadecene (ODE, 90%), oleic acid (OA, 90%), sodium oleate (NaOL, 90%), and 4-vinylbenzoic acid (97%) were purchased from Sigma-Aldrich. The solvents acetone, chloroform, diethyl ether, methanol, and toluene were obtained from different suppliers and had a minimum purity of 99%. Additionally, diethyl ether was distilled before use.

### Dynamic Light Scattering (DLS)

DLS measurements were conducted using a Zetasizer Nano ZS (Malvern Panalytical) and analyzed with the Zetasizer Nano ZS software 8.00.4813 (Malvern Panalytical) or using a Zetasizer Pro Blue equipped with the software ZS XPLORER 3.2.0.84 (Malvern Panalytical). For sample preparation, the dispersion (5–10 μL) was diluted with toluene (2 mL, isolated nanoparticles) or ultrapure water (2 mL, SPs) yielding a faint brownish coloration. Measurement parameters were selected automatically by the device. Each sample was measured three times with a minimum of 11 scans per measurement.

### Zeta Potential Measurements

The zeta potential of encapsulated SPs was measured using a Zetasizer Pro-Blue equipped with the software ZS XPLORER 3.2.0.84 (Malvern Panalytical). The sample (10 μL) was diluted with KCl solution (1 mL, 0.1 M). The pH of the dispersions was adjusted to 8.1 before the measurement.

### Transmission Electron Microscopy (TEM)

TEM images were recorded using a JEM-1011 electron microscope (JEOL, 100 kV). Samples were prepared by dropping diluted dispersions of the particles onto 400 mesh carbon-coated TEM copper grids.

### Thermogravimetric Analysis (TGA)

TGA was performed using a NETZSCH TG 209 F1 Libra and the software NETZSCH Measurement, version 8.0.3. The samples (minimum 3 mg) were filled into an alumina crucible. The TGA was conducted from 25 to 800 °C with a heating rate of 10 K/min and a nitrogen flow of 60 mL/min. The organic fraction, used to normalize the VSM curves to the inorganic mass, was determined after reaching a temperature of 600 °C.

### Fourier Transform Infrared (FTIR) Spectroscopy

FTIR measurements were performed using a Bruker Invenio R spectrometer. For preparation, the sample (2 mg) was magnetically separated and the resulting pellet was dried. Samples that could not be magnetically separated (individually encapsulated SPIONs and SPs treated with a 30-fold higher DTAB concentration) were dried directly from the dispersion. Afterward, the obtained powder was mixed with KBr and ground until the powder showed a faint color. KBr FTIR spectra were measured in the wavenumber range between 370 and 3500 cm^–1^ with a resolution of 4 cm^–1^ in transmittance. The software OPUS 8.1 was used to record the spectra.

### Scanning Electron Microscopy (SEM)

SEM images were recorded using a Leo 1550 (GEMINI) with acceleration voltages set in the range between 0.1 and 30 kV. For sample preparation, a diluted dispersion of the particles was dropped onto a silicon wafer and dried.

### Small-Angle X-ray Scattering (SAXS)

SAXS measurements were conducted using a self-designed apparatus with an Incoatec X-ray source IμS with Quazar Montel optics, and a CCD detector Rayonix SX165. Between sample and detector, an evacuated flight tube with a distance of 1.1 m was installed. The focal spot size had a diameter of 700 μm at a wavelength of 0.1542 nm. For the sample preparation, 20 μL of the aqueous SP dispersion (concentration: ∼10 mg/mL) was dropped onto a piece of Kapton foil and dried. The regular measurement time was accounted for 10 min per sample. As a control software, SPEC (version 5.32, Certified Scientific Software, Cambridge, MA, U.S.A.) was employed. Data reduction to 1D scattering curves was done by DPDAK (version 1.5.0).^38^ The obtained data from spherical SPIONs were fitted using the software Scatter, version 2.5.^39,40^ The evaluation of SAXS curves of SPs based on cubic SPIONs was performed using the software OriginPro 2019, version 9.6.0.172. The scattering vector *q* was determined using the peak analysis tool. To determine the interparticle distances within the SPs, the lattice parameter was calculated and the edge length of SPIONs (determined by TEM) was subtracted. Additional SAXS measurements were performed at beamline P03 (PETRA III, DESY, Hamburg, Germany).^41^ For the measurements, the X-ray wavelength was fixed at 1.044 Å with beam dimensions of ∼25 × 30 μm^2^ (vertical × horizontal direction). The scattering patterns were obtained using a Pilatus 2 M detector (Dectris) with a sample-to-detector distance of roughly 5.540 ± 0.005 m. All samples (concentration: ∼20–30 mg/mL in an aqueous DTAB solution of 4.5 mg/mL) were filled into quartz glass capillaries, sealed, and scanned over a height of 2 mm with a step size of 0.1 mm. For the data reduction of the 2D-SAXS data, similar images for each sample were summed up and azimuthally averaged using numerical recipes and scaling procedures from literature.^42^

### Vibrating Sample Magnetometer (VSM)

The magnetic properties were measured using a VSM EZ-9 from MicroSense. The sample (55 μL) was filled into a 6 mm poly(ether imide) “ultem” cup. The measurement range was set between −2.5 and 2.5 T with a step size of 100 mT between 2.5 and 0.5 T, 10 mT between 0.5 and 0.1 T and 1 mT steps between 0.1 and −0.1 T.

### X-ray Diffraction (XRD)

XRD measurements were conducted using an X’Pert Pro diffractometer from PANalytical. The nanoparticle sample (∼20 mg) was dried, ground, and placed on a (911) silicon wafer. XRD patterns were measured between 10° and 90° on the 2θ scale.

### Syntheses of Iron Oxide Nanocubes and Nanospheres

Cubic SPIONs were synthesized according to the protocol of Kampferbeck et al.,^43^ which is based on previous studies of Yu et al.^44^ and Li et al.^45^ Spherical SPIONs were synthesized according to Yu et al.^44^ A detailed description of the syntheses and the sample characterization (DLS, TEM, and TGA) can be found in section 1 of the Supporting Information.

### Synthesis of 11-(2-Bromoisobutyrate)-undecyl-1-phosphonic Acid (BiB-UDPA)

BiB-UDPA was purchased from Sigma-Aldrich or synthesized as previously described by Kampferbeck et al.^21^ following the approach by Minet et al.^28^

### Synthesis of *N*,*N*-Bis(2-pyridylmethyl)octadecylamine (BPMODA)

BPMODA was synthesized according to Kampferbeck et al.^21^ following the approach described by Menger and Lee.^46^

### Synthesis of 4-Vinylbenzyl Azide

4-Vinylbenzyl azide was synthesized according to Albuszis et al.^47^ Details of the synthesis can be found in section 2 of the Supporting Information.

### Assembly of Supraparticles (SPs) and Ligand Exchange (LE)

The SPs were assembled following the procedure reported by Paquet et al.^12^ and Cao and co-workers.^48^ In short, SPIONs (10 mg, mass including OA ligands) were dispersed in 1 mL chloroform and mixed with aqueous DTAB solution (1.0 mL, 4.5–150 mg/mL) using a syringe under stirring on a vortex mixer (3500 rpm) and then stirred for 30 s. The resulting light brown emulsion was transferred to a beaker and mechanically stirred for 60 min and at 300 rpm at room temperature using the stirrer of the EasyMax 402 (Mettler Toledo). DLS measurements were done to determine the size and polydispersity index (PDI) of the SPs (note: Complete removal of the chloroform is indicated by constant PDIs obtained from DLS measurements). Subsequently, the SP dispersion was centrifuged at 3500 g for 5 min. The pellet was collected and redispersed in an aqueous DTAB solution (1.0 mL, 4.5 mg/mL) to obtain a concentration of roughly 10 mg/mL. After optimization of the process, DTAB concentrations of 20 mg/mL were used to conduct the EISA.

Ligand exchange with BiB-UDPA was conducted in an emulsion system. To this end, the required amount of BiB-UDPA was roughly calculated as detailed in section 3 of the Supporting Information. Briefly, BiB-UDPA (0.093 mg, 0.23 μmol) was dissolved in chloroform (0.5 mL). The solution was added to the SP dispersion (1.0 mL, 10 mg/mL), shaken, and stirred on a lab shaker (KS-10 swip, Edmund Bühler) at 200 rpm for 45–60 min. Additionally, the emulsion was shaken by hand in roughly 10–15 min time intervals to avoid the separation of water and oil phases. Afterward, the SP dispersion was transferred into a reactor (EasyMax 402, Mettler Toledo) and diluted with DTAB (12–15 mL, 4.5 mg/mL) to obtain a concentration of about 0.7–0.8 mg/mL. The dispersion was equilibrated at 300 rpm for 45 min at room temperature.

For assembly of 20 mg SPIONs into SPs, 1 mL of a SPION dispersion (concentration 20 mg/mL) was injected into 1 mL DTAB (20 mg/mL) during vortexing using a syringe. After 30 s vortexing, the emulsion was transferred to a beaker and stirred mechanically for another 60 min at 300 rpm. Afterward, the emulsion was purged with nitrogen for 15 min under continuous stirring at 300 rpm. After centrifugation at 3000 g for 5 min, the particles were dispersed in aqueous DTAB solution (1.0 mL, 4.5 mg/mL) to obtain a concentration of roughly 20 mg/mL. Subsequently, BiB-UDPA (0.170 mg, 0.42 μmol) was dissolved in chloroform (0.5 mL). Otherwise, the ligand exchange was conducted as described above.

### Encapsulation and Surface Functionalization of Supraparticles via AGET ATRP

The ATRP protocol is based on the approach of Kampferbeck et al.^21^ The SP dispersion obtained after LE with BiB-UDPA (concentration 0.7–0.8 mg/mL) was heated to 70 °C (jacket temperature) in the reactor (EasyMax 402, Mettler Toledo) and purged with nitrogen for 15 min to remove chloroform and oxygen from the dispersion. Meanwhile, the CuBr_2_/BPMODA (*N*,*N*-bis(2-pyridylmethyl)octadecylamine) complex was produced. BPMODA (32 mg, 71 μmol) and CuBr_2_ (7.0 mg, 31 μmol) were dispersed in THF (1.0 mL) and dried. The catalyst was redispersed in THF (1.0 mL) and the dispersion was divided in eight parts and dried. The dried CuBr_2_/BPMODA complex was stored at −18 °C. For the encapsulation of SPs with different monomer volumes, one catalyst portion, corresponding to 4.9 mg CuBr_2_/BPMODA was dispersed in a mixture of styrene and divinylbenzene (DVB), as reported previously.^49,50^ The overall volume of the styrene/DVB mixture (1:1, v/v) was varied between 70 and 280 μL. SPs prepared for SAXS measurements on Kapton foil and SPs based on differently sized SPIONs were encapsulated using only half a portion (2.5 mg) of the catalyst. The nitrogen inlet to the SP dispersion was stopped, and the catalyst complex was injected to the SP dispersion. The stirring speed was increased to 500 rpm and the dispersion was heated to 65 °C. After 5 min, a nitrogen saturated aqueous ascorbic acid solution (1.4 mL, 5 mM) was added and the stirring speed was decreased to 300 rpm. For encapsulations using one-half of the catalyst, only 700 μL ascorbic acid was added. The polymerization was stopped after 6 h by cooling the reaction to room temperature under ambient atmosphere. The polymer-encapsulated SPs were magnetically separated using a NdFeB cuboid magnet (N40, holding force about 100 kg)^51^ and the collected pellet was redispersed in aqueous DTAB solution (1.0 mL, 4.5 mg/mL). This purification step was conducted twice.

To decorate the polymer shell with functional groups, the CuBr_2_/BPMODA catalyst was dispersed in DVB (70 μL, 0.48 mmol), styrene (57 μL, 0.50 mmol), and 4-vinylbenzyl azide (13 μL, 0.099 mmol) or 4-vinylbenzoic acid (13 mg, 0.088 mmol). Apart from this, the same protocol as described for unmodified polymers shells was used.

The encapsulation of 20 mg assembled SPIONs was conducted using a total of 140 μL monomer (styrene/DVB). Otherwise, the procedure was unchanged. The final concentration of the SPs was 12 mg/mL.

### Preparation of Individually Encapsulated SPIONs

The encapsulation of individual SPIONs using AGET ATRP was carried out according to the approach of Kampferbeck et al.^21^ The final concentration was 5.1 mg/mL.

## Results and Discussion

### Strategy for the Polymer Encapsulation of Supraparticles

**Figure 1:**
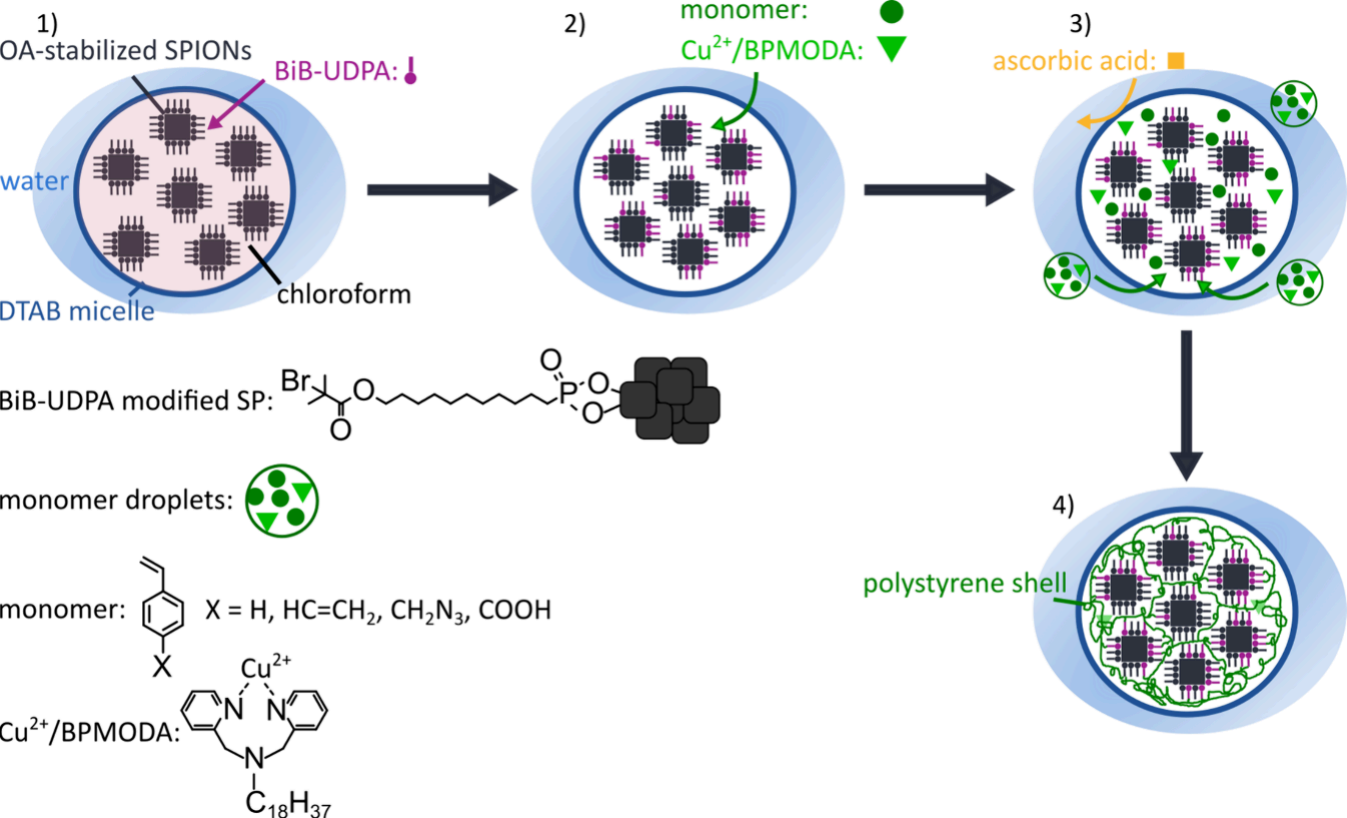
Strategy for the surface-grafted polymer encapsulation of SPs from SPIONs: (1) Ligand exchange of oleic acid (OA) for BiB-UDPA. (2) Addition of the catalyst Cu^2+^/BPMODA and the monomer [e.g., styrene and divinylbenzene (DVB)]. (3) Reduction of the catalyst and initiation of the ATRP using ascorbic acid. (4) Controlled ATRP growth of the polymer shell.

Figure 1 illustrates the proposed strategy for the synthesis of surface-grafted, polymer-encapsulated SPs from cubic SPIONs. The process starts with SPs prepared from oleic acid (OA) stabilized SPIONs, which were assembled into SPs using the evaporation induced self-assembly (EISA) process.^12,37^ The SPs are stabilized with a dodecyltrimethylammonium bromide (DTAB) surfactant shell and are dispersed in water. First, OA ligands on the SPION surface are exchanged for the atom transfer radical polymerization (ATRP) initiator ligand 11-(2-bromoisobutyrate)-undecyl-1-phosphonic acid (BiB-UDPA) using chloroform as solvent. Due to the high affinity of the phosphonic acid group to iron oxide surfaces, it is expected that the BiB-UDPA ligands bind preferentially to the SPIONs at the SP surface.^31−33^ After solvent evaporation, the monomers [e.g., styrene and divinylbenzene (DVB)]^19,49,52^ and the activators generated by electron transfer^26,27^ (AGET) catalyst Cu^2+^/BPMODA (*N*,*N*-bis(2-pyridylmethyl)octadecylamine) are added. These compounds diffuse into the SP micelles or form monomer droplets in the aqueous phase. Afterward, the reducing agent ascorbic acid is added to initiate the polymerization. Due to its hydrophilic character, it is expected that ascorbic acid reduces the Cu^2+^ catalyst preferentially at the SP/water interface. After the initiation, the polystyrene shells are grown via the controlled ATRP reaction. The proposed mechanism of the AGET ATRP is shown in Figure S3 in section 4 of the Supporting Information.

### Synthesis of Supraparticles and Ligand Exchange

Cubic SPIONs with edge lengths ranging from ∼12 to ∼30 nm were used to synthesize spherical SPs via the EISA process.^12,37^ For comparative SAXS measurements, SPs were synthesized from ∼12 nm sized spherical nanoparticles using the same approach.

**Figure 2:**
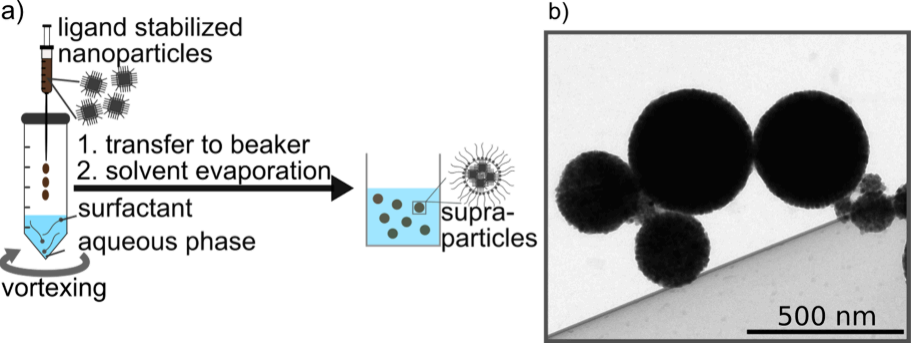
(a) Scheme of the modified EISA process using the “syringe injection method”. (b) Representative TEM image showing SPs synthesized with the optimized DTAB concentration of 20 mg/mL.

In the EISA process, a hydrophobic nanoparticle solution is mixed with an aqueous surfactant solution to produce an oil-in-water emulsion, in which the nanoparticles are dispersed in surfactant-stabilized oil droplets. Subsequently, the hydrophobic solvent is evaporated and the nanoparticles agglomerate inside the shrinking oil droplets. Finally, the resulting nanoparticle clusters form micellar structures with the surfactant molecules in the aqueous phase. Here, we combined the EISA approaches reported by Paquet et al.^12^ and Zhuang et al.,^48^ as schematically shown in Figure 2a. The size and size distribution of the SPs was optimized using different DTAB concentrations and the “syringe injection method” (cf. Figure 2a). The method and the results of the optimization process are described in section 5 of the Supporting Information. On the basis of these results, we synthesized all SP samples for subsequent experiments using 1.0 mL of an aqueous dodecyltrimethylammonium bromide (DTAB, 20 mg/mL) solution and 1.0 mL of oleic acid (OA) coated SPIONs dispersed in chloroform (10 mg/mL). Unless stated otherwise cubic SPIONs with an edge length of 15.1 ± 1.5 nm were used for the self-assembly into SPs. The TEM image shown in Figure 2b confirms the formation of spherical SPs with diameters of ∼200 to ∼400 nm using the optimized protocol. Due to their large size, the obtained SPs showed high magnetophoretic mobility and, thus, fast responses to external magnetic fields.

After preparing the SPs, their encapsulation was performed via AGET^26,27^ ATRP.^14,20^ To this end, we used the ATRP initiator ligand 11-(2-bromoisobutyrate)-undecyl-1-phosphonic acid (BiB-UDPA), which was previously used by our group for the AGET ATRP encapsulation of individual SPIONs.^21^ In our previous study, the exchange of the initial ligand OA for the ATRP initiator ligand was conducted in chloroform. In our present study, the ligand exchange (LE) reaction was conducted in the emulsion system. We expected that the BiB-UDPA ligands, dissolved in chloroform, diffuse into the DTAB micelles and replace the initial OA ligands on the surface of the clustered nanocubes. The molecular structure of the surface-grafted BiB-UDPA ligand is shown in Figure 1. The LE reaction is known to proceed rapidly due to the phosphonic acid anchoring group, which ensures the efficient replacement of the initial OA ligands on the iron oxide surface.^31−33^ Further, we assumed that the BiB-UDPA ligands bind preferentially to nanocubes located close to the SP surface. Therefore, the amount of added initiator molecules corresponded roughly to the number of ligands needed to cover the surface of the SPs with one ligand monolayer. Details of this calculation can be found in section 3 of the Supporting Information.^21^

**Figure 3:**
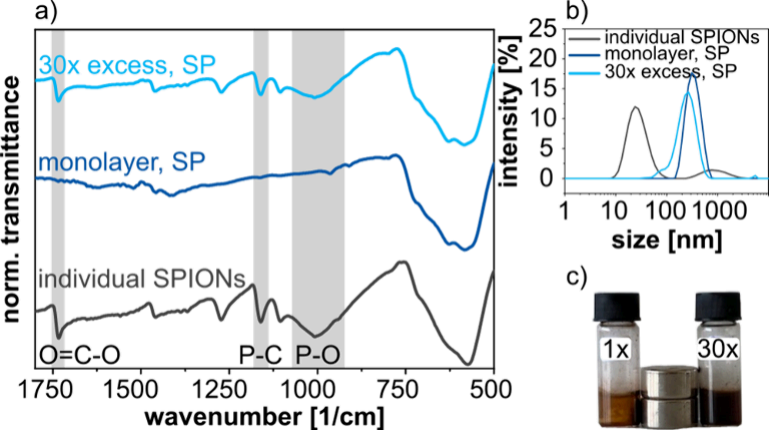
(a) FTIR spectra of individual SPIONs (edge length ∼ 14 nm) after surface modification with BiB-UDPA ligands (black curve), SPs based on 12 nm cubic SPIONs treated with an amount of BiB-UDPA ligands corresponding to roughly a monolayer coverage of the SPs (dark blue line), and of SPs treated with a 30-fold higher concentration of BiB-UDPA (light blue line). (b) Corresponding DLS size distributions of individual SPIONs and SPs after the reaction with BiB-UDPA corresponding to either a monolayer or a 30-fold higher concentration. (c) Photography of the SP dispersions with 1× and 30× concentration of BiB-UDPA under the influence of two NdFeB disk magnets (N42).^54^ The concentration of SPs in both samples was identical (10 mg/mL).

Figure 3a presents the Fourier transform infrared (FTIR) spectrum of the surface-modified SPs based on ∼12 nm cubic SPIONs (dark blue line). This spectrum is compared to the spectrum of individual cubic SPIONs of ∼14 nm edge length modified with the same BiB-UDPA ligand (black line), reproduced from Kampferbeck et al.^21^ The broad band between 1100 and 900 cm^–1^ corresponds to the P–O stretching vibration and indicates the successful LE reaction.^53^ However, this band is not clearly visible in the spectrum of the modified SPs. We attribute this finding to the low concentration of the added BIB-UDPA ligand. Therefore, the LE reaction was repeated with a 30-fold increased BiB-UDPA concentration but otherwise unchanged parameters (light blue curve). This time, the characteristic vibration is clearly visible, confirming the successful LE reaction in the emulsion system. Additional characteristic bands supporting the successful ligand exchange are observed at 1731 and 1160 cm^–1^. We attribute these bands to the BiB-UDPA ligand ester carbonyl and P–C stretching vibration, respectively.^21,53^ Also the other bands match the IR signature of BiB-UDPA bound to SPIONs.^21^

Additionally, we observed differences in the magnetic behavior of the SPs treated with the 30-fold excess of BiB-UDPA or with the concentration corresponding to one monolayer coverage. In the former case, a large amount of SPIONs could not be separated from the dispersion using two NdFeB disk magnets (N42, ∼ 3.7 kg holding power; Figure 3c), indicating the presence of nonaggregated SPIONs or SP fragments. In contrast, the SPs treated with the lower BiB-UDPA concentration could be separated much more efficiently. We conclude that the excess of the ATRP ligand degrades the stability of the DTAB-stabilized micellar structures. Thus, fragmented SPs were obtained, which could not be separated by magnetic forces (cf. Figure S5 of the Supporting Information). Note, the DLS intensity distribution of the SPs prepared with the 30-fold excess of BiB-UDPA shown in Figure 3b) confirms the presence of fragmented SPs or individual SPIONs. The whole distribution is shifted to smaller sizes and a shoulder revealing the presence of SP fragments with sizes below 100 nm is clearly recognized. However, the differences appear rather small due to the much stronger scattering of larger particles that superposes the weaker signal of the smaller nanoparticles. Further, the DLS curve of the individual SPIONs (gray curve) shows a small fraction of aggregated SPIONs.

In conclusion, the exchange of OA ligands for BiB-UDPA ligands on the surface of aggregated cubic SPIONs is feasible in the emulsion system. Further, it is necessary to avoid too high concentrations of the BiB-UDPA ligand to preserve integrity of the SPs.

### Encapsulation and Functionalization of Supraparticles

After exchanging the initial OA ligands for BiB-UDPA, the SPs were encapsulated via surface grafted AGET^26,27^ ATRP.^14,20^ To this end, the BIB-UDPA-modified SPs were diluted with DTAB solution. The final concentration of SPs was ∼0.7–0.8 mg/mL. Subsequently, chloroform and oxygen were removed from the solution by purging with nitrogen and heating the dispersion to 65 °C. The CuBr_2_/BPMODA catalyst was dissolved in the desired monomer volume and added to the dispersion. Finally, the ATRP was initiated via addition of ascorbic acid as reducing agent (cf. Figure 1). Since magnetic stirring of the reaction mixture leads to unwanted precipitation of superparamagnetic SPs, the ATRP was conducted in a mechanically stirred reactor as detailed in the Experimental Section.

**Figure 4:**
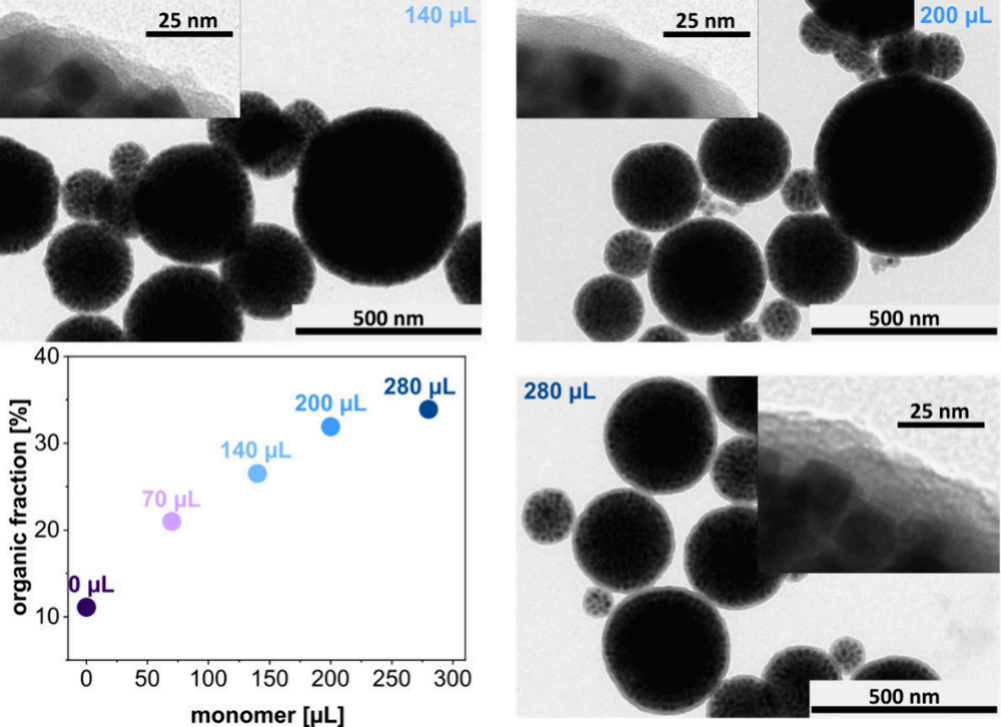
TEM images of encapsulated SPs. The images were taken after magnetic purification and reveal an increasing polymer shell thickness with increasing volume of the added styrene/DVB mixture (140, 200, and 280 μL). The TGA data (lower left panel) confirm an increasing organic fraction of the encapsulated SPs with increasing volume of added monomers.

**Figure 5:**
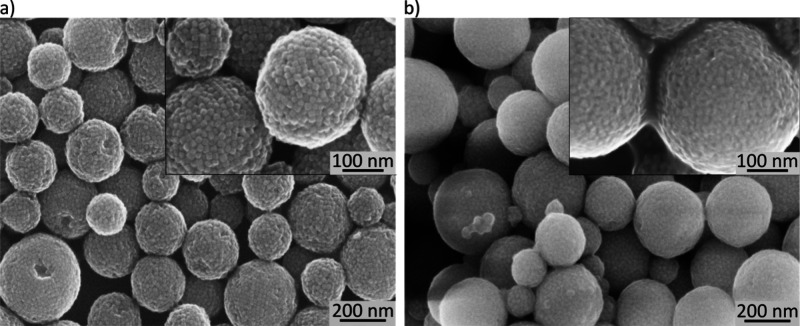
(a) SEM images of SPs after centrifugal purification and before polymer encapsulation. Some SPs show ordered domains of nanocubes. (b) SPs after polymer encapsulation and magnetic purification. A mixture of 100 μL styrene and 100 μL DVB was used to conduct the AGET ATRP reaction.

In order to obtain a cross-linked polystyrene shell, a monomer mixture of styrene and divinylbenzene (DVB) was used for conducting the ATRP. This approach has been reported before to obtain branched polystyrene.^49,50^ In our approach, the shell thickness could be adjusted by varying the overall volume of the added styrene/DVB mixture (70–280 μL), while keeping all other parameters constant. Figure 4 presents TEM images of the encapsulated SPs after magnetic purification. Additional TEM images of the SPs encapsulated with 70 μL and the TGA data of the samples can be found in Figure S6 in section 6 of the Supporting Information). The data confirm an increasing PS shell thickness with increasing volume of added styrene/DVB. Further, the SEM images presented in Figure 5 reveal the typical surface morphology of the SPs before and after polymer encapsulation. To study the stability of the as assembled SPs and the SPs after encapsulation, we stored the samples for at least 6 months in dispersion. TEM images after this time interval show that both SP samples retained their original shape, indicating long-term stability (Figure S7 of the Supporting Information).

In agreement with the TEM characterization, the results of TGA measurements shown in Figure 4 confirm an increasing organic fraction of the encapsulated SPs with increasing volume of the added monomer (styrene/DVB). The mass fraction increases linearly between 70 and 200 μL of added monomer. Bourgeat-Lami and co-workers observed similar trends for the conversion in a seeded emulsion macroRAFT polymerization of block copolymers used as stabilizers for iron oxide SPs.^19^

The SPs encapsulated using different monomer volumes were also characterized by dynamic light scattering (Figure S8 of the Supporting Information). To this end, the intensity weighted size distributions of the SPs were measured prior and after the encapsulation. Results shown in Table S2 of the Supporting Information indicate PDI values between 0.1 and 0.2 prior to encapsulation. After polymerization, the PDI increased to values between 0.2 and 0.4. Additionally, we observed an increase of the *z*-average values, especially pronounced after the magnetic separation following the encapsulation. These observations indicate the tendency of the encapsulated SPs to aggregate during magnetic separation.

Exemplarily, we calculated the yield of the SPs encapsulated using a monomer volume of 140 μL. On the basis of TGA data, 53% of the initial SPION mass was recovered after polymer encapsulation and magnetic separation of the SPs.

**Figure 6:**
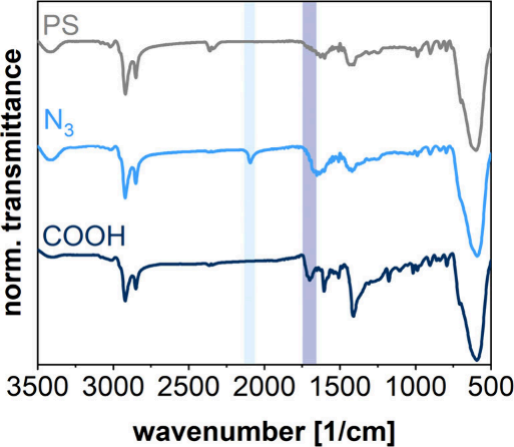
FTIR spectra of SPs with functionalized and nonfunctionalized polystyrene (PS) shells, as indicated. Characteristic vibrations expected for carboxylic acid (COOH) and azide (N_3_) functional groups are indicated by dark blue and light blue bars, respectively.

To introduce functional groups into the polymer shell, we substituted 9% (v/v) of styrene for functionalized monomers, i.e., vinylbenzyl azide^47,55^ or vinyl benzoic acid.^11,56^ This approach has been reported in several previous studies.^57^ As indicated in Figure 6, FTIR spectra of the prepared polymer-encapsulated SPs confirmed the incorporation of azide and carboxylic acid groups. The characteristic N=N stretching vibration of the azide group (light blue highlighted area) appears at 2093 cm^–1^.^58^ For the carboxylic acid modified SPs, the band at 1701 cm^–1^ corresponds to the C=O stretching vibration (dark blue highlighted area).^59^ Weak aromatic C–H stretching vibrations at 3060 and 3026 cm^–1^ as well as methylene group vibrations at 2920 and 2850 cm^–1^ are attributed to polystyrene.^60^ Further, zeta potential measurements at pH 8.1 revealed a zeta potential of polystyrene encapsulated SPs of −3.4 ± 2 mV. This value decreased to −15.1 ± 2 mV for carboxylic acid modified SPs, supporting the incorporation of negatively charged carboxylate groups within the polymer shell.

### Arrangement of Nanocrystals within Supraparticles

**Figure 7:**
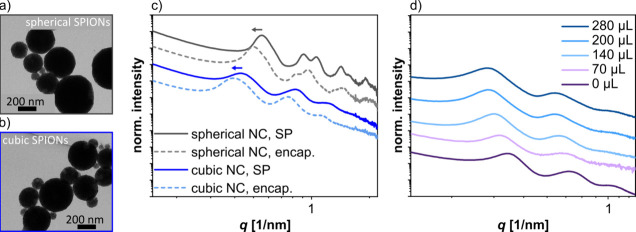
(a) TEM images of SPs from ∼12 nm sized spherical nanocrystals and (b) cubic nanocrystals. (c) SAXS curves of SPs from spherical (gray curves) and cubic (blue curves) SPIONs measured before (solid lines) and after (dashed lines) the encapsulation using 70 μL monomer (styrene/DVB). The arrows indicate the shift of the SAXS signals after the encapsulation. The data was measured using dried SPs on Kapton foil. (d) SAXS curves of dispersed SPs in DTAB solution before the encapsulation (0 μL) and after encapsulation using monomer volumes between 70 and 280 μL, as indicated.

The arrangement of the nanocrystals within the SPs before and after polymer encapsulation was studied by small-angle X-ray scattering (SAXS). Respective SAXS curves of SPs assembled from both, spherical and cubic SPIONs are shown in Figure 7c. These samples were dried on Kapton foil for conducting the SAXS measurements. For SPs based on spherical SPIONs, the data are consistent with a face-centered cubic (fcc) superlattice. Details of the data analysis, including the values of various parameters (lattice constant, nearest neighbor distance, nanocrystal radius, and domain sizes) extracted from the SAXS curve fits, can be found in Figure S9 and Table S3 of the Supporting Information. The nanosphere size of nonencapsulated SPs was 12.6 nm in reasonable agreement with their mean diameter of 11.9 ± 0.70 nm determined by TEM (section 1 of the Supporting Information). Further, the nearest neighbor center-to-center distance of the nanocrystals within the nonencapsulated SPs was 13.8 nm. Subtracting the nanocrystal size (determined by SAXS) returns edge-to-edge interparticle distances of 1.2 nm, indicating interdigitation or bending of the OA ligands.^61^ After the polymer encapsulation (dashed curves), the reflections in the SAXS curves (Figure 7c) shifted to smaller scattering vectors, revealing increased interparticle distances of 2.4 nm. The fcc lattice constant increased from 19.5 to 21.5 nm. We attribute this increase of the interparticle distances to swelling based on partitioning of monomer molecules into the ligand matrix during the encapsulation process.

For cubic SPIONs, however, it was more challenging to produce suitable fits to the SAXS data using the standard lattice models. Hence, we calculated the lattice constant *L* based on the scattering vector *q* of the first reflection by assuming a simple cubic lattice (section 7 and Figure S9 of the Supporting Information). The *q* values for SPs from the same cubic SPIONs were 0.428 and 0.394 nm^–1^ before and after polymer encapsulation, respectively. These *q* values correspond to lattice constants *L* of 14.7 and 15.9 nm, respectively. Subtracting the cubes’ edge length *s* obtained from TEM images (12.2 ± 1.4 nm) returned an approximate edge-to-edge distance of 2.5 and 3.7 nm, respectively. These edge-to-edge distances are larger than those in SPs from spherical SPIONs (1.2 and 2.4 nm, respectively). We assume that the increased edge-to-edge distances are due to increased standard deviations of the nanocube sizes and more dense initial ligand coverages compared to spherical SPIONs (cf. section 1 of the Supporting Information). Further, SAXS curves of SPs from spherical SPIONs show more pronounced higher orders of reflections, indicating a higher degree of nanocrystal ordering (cf. gray curves in Figure 7c). Consistent with this observation, the SPs assembled from spherical nanocrystals often showed a faceted surface morphology in contrast to the SPs from nanocubes (cf. TEM images presented in panels a and b of Figure 7). We assume that the narrow size distribution of the spherical SPIONs promoted the formation of highly ordered and densely packed SPs.

To further investigate swelling of the SPs during polymer encapsulation, we measured SAXS of SPs from cubic SPIONs with a mean edge length of 15.1 ± 1.5 nm. These measurements were performed at the PETRA III (DESY) light source with the SPs dispersed in an aqueous DTAB solution (4.5 mg/mL). Here, we compared samples before and after polymer encapsulation, using monomer volumes from 70 to 280 μL. As seen in Figure 7d, the increase of the monomer volume resulted in an increasing shift of the SAXS reflections to smaller scattering vectors. As for the ∼12 nm sized cubic SPIONs (see above), we calculated the approximate edge-to-edge distances between the nanocubes based on the scattering vector of the first reflection (Figure S10 and Table S4 of the Supporting Information) and the average edge length determined by TEM. The approximate edge-to-edge distance increased with increasing monomer volume from 1.4 nm (before encapsulation) to 5.0 nm (280 μL monomer). In summary, this finding shows that beside the shell thickness of the SPs (cf. Figure 4) the distance between the clustered nanocrystals can be adjusted by varying the amount of the monomer added for the polymer encapsulation.

### Superparamagnetic Properties of SPs

In comparison to individually encapsulated SPIONs, the prepared SPs showed much faster responses to external magnetic fields. Such fast responses enable the application as magnetic carriers.^3,19^

To further investigate the SPs’ superparamagnetic properties, we assembled 20 mg SPIONs and encapsulated them within 140 μL monomer. VSM curves of encapsulated SPs based on ∼15 nm sized cubic SPIONS and individually encapsulated SPIONs of the same batch were measured. Results shown in Figure S11 in section 8 of the Supporting Information indicate the same magnetic behavior in both samples. Further, absence of hysteresis confirms that the superparamagnetism of the individual SPIONs was preserved after assembling them into SPs.

**Figure 8:**
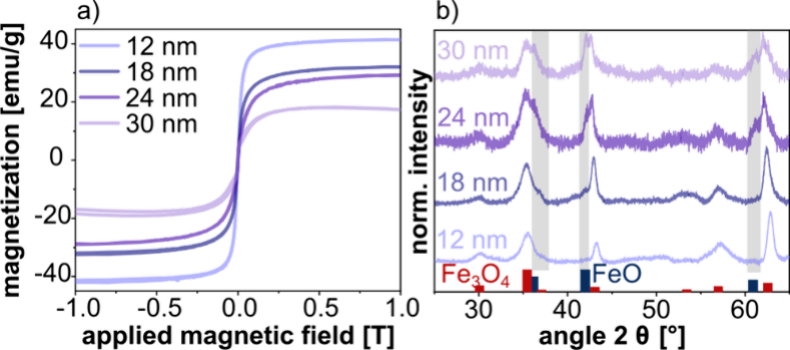
(a) Magnetization curves of SPs based on 12 (light blue curve), 18 (dark blue curve), 24 (dark purple curve) and 30 nm (light purple curve) sized nanocubes obtained from VSM measurements. The data were normalized to the inorganic mass fraction of the nanocomposites. (b) Corresponding powder X-ray diffraction measurements of 12, 18, 24, and 30 nm sized cubic nanocrystals. Gray highlighted areas indicate the position of wustite peaks. The magnetite (red, AMCSD code: 0017981) and wustite (dark blue, AMCSD code: 0018069) reference data were taken from refs (65) and (66), respectively.

It is well-known that the phase composition and thus the magnetization of SPIONs depends on the nanocrystal size.^62,63^ Usually, SPIONs synthesized via thermal decomposition of iron oleate are initially formed in the wustite modification and oxidize subsequently to magnetite/maghemite when stored under air.^62−64^ However, if the SPIONs exceed a size of about 18 nm, the oxidation process is significantly retarded and a wustite core remains within the SPION. Since wustite is an antiferromagnetic material, lower magnetizations have been observed for SPIONs with increasing size.^62^ To study this effect, we measured the magnetization of the SPs from nanocubes with edge lengths of ∼12, ∼18, ∼24, and ∼30 nm. Parameter settings for the preparation and encapsulation of the SPs were unchanged for all four samples. The monomer volume was 70 μL (styrene/DVB) for the encapsulation of 20 mg SPs. Aqueous DTAB dispersions of the SPs were used for the VSM measurements. Figure 8a presents the magnetization curves which were normalized to the inorganic fraction of the samples. TEM images of the SPs are presented in Figure S13 of the Supporting Information. Figure S14 of the Supporting Information shows the VSM data normalized to the total mass of the nanocomposite and the respective TGA data.

The VSM curves of all SP samples reveal superparamagnetic behavior. Moreover, the saturation magnetization decreases with increasing nanocube sizes. The highest magnetization (40 emu/g) was measured for the sample prepared from 12 nm cubes, while the lowest magnetization (∼15 emu/g) was obtained for the sample with 30 nm cubes.

The powder XRD data of the nanocubes used for SP preparation are presented in Figure 8b. The data for the 12 nm nanocube sample clearly reveal the magnetite/maghemite modification (note: only the magnetite reference is shown in Figure 8b because the diffraction pattern of maghemite is very similar).^67^ As expected, the characteristic wustite reflexes appear with increasing intensity as the size of the nanocubes increases. Hence, in agreement with previous studies, the decreasing saturation magnetization of our SPs formed from nanocubes with increasing sizes is attributed to an increasing mass fraction of the antiferromagnetic wustite core.

## Conclusion

In this study, we presented a surface grafted AGET ATRP synthesis for the encapsulation of iron oxide SPs within a cross-linked polystyrene shell. Compared to previous work, the presented approach accelerates the formation of SPs and utilizes a well-controlled radical polymerization reaction, yielding encapsulated SPs with a smooth polymer shell of tunable thickness. The SPs were assembled from cubic SPIONs using the EISA process and subsequently modified with the ATRP initiator ligand BiB-UDPA. After addition of the catalyst/monomer mixture, the growth of the polymer shell was started by reducing the Cu^2+^ catalyst with ascorbic acid. The obtained SPs were characterized using DLS, TEM, SEM, TGA, FTIR, SAXS, VSM, and XRD.

The thickness of the polymer shell could be adjusted in the range of several nanometers by varying the added monomer volume. Further, by adding functionalized styrene monomers to the ATRP reaction it was possible to modify the polymer shell with azide and carboxylic acid groups. The presence of these functional groups was confirmed using FTIR and zeta potential measurements. Azide groups can be transferred into amine groups via Staudinger reduction^68^ or hydrogenation^68^ and used in a broad variety of biochemical applications, e.g., chemical/biological conjugation.^69^ Further, the use of aqueous dispersions reduces the amount of organic solvents and offers effective heat-dissipation during the polymerization.^23^ Additionally, our materials show high magnetophoretic mobilities and long-term-stability, especially for the encapsulated SPs, which maintained their shape over at least 33 months. Therefore, they can be reused several times, e.g., for magnetic separations, avoiding waste production. However, future experiments should focus on the optimization of the polymerization, e.g., increasing the monomer conversion and minimizing the catalyst amount.^70^

SAXS measurements revealed an ordered arrangement of SPIONs within the SPs. However, compared to SPs from spherical SPIONs, the degree of ordering was less pronounced in the case of cubic SPIONs, which is possibly due to increased size and shape deviations of the nanocubes and the misalignment of neighboring nanocube layers. Furthermore, the SAXS curves shifted to lower scattering vectors during polymer encapsulation, revealing the diffusion of monomer into the SPs’ interior structure.

VSM measurements revealed the same superparamagnetic behavior of SPs and the nanocube building blocks. Additionally, the VSM curves showed the same saturation magnetization as the individual SPIONs after normalizing the curves to the inorganic mass. However, since the magnetophoretic mobility of SPs is much higher than of individual nanocubes, the SPs could be separated from dispersion via magnetic forces much more efficiently than individually encapsulated SPIONs. As an additional finding, we observed that the saturation magnetization of the SPs decreased when increasing the nanocube size from 12 to 30 nm. This effect is caused by an increasing fraction of the antiferromagnetic wustite phase with increasing nanocube size.

In conclusion, the AGET ATRP-based strategy presented in this study provides an interesting route to the well-controlled polymer encapsulation of SPs from SPIONs. The thickness of the smooth polymer shell as well as the interparticle spacing can simply be adjusted by varying the amount of added monomer. Using different styrene derivatives for the polymerization provides an efficient approach to surface-functionalized superparamagnetic SPs which are of great interest for a broad variety of biomedical applications.

## Supporting Information Available

Additional experimental details and further figures and graphs to support the presented data in the main document, including the synthesis of SPs, the encapsulation, the SAXS analysis, the magnetic properties, the synthesis of SPIONs and monomers, and the calculation of the required mass of BiB-UDPA (PDF)
