# Supplementary material for: Supraparticles from Cubic Iron Oxide Nanoparticles: Synthesis, Polymer Encapsulation, Functionalization, and Magnetic Properties
Source: Langmuir. 2024 Oct 18;40(43):22762–72. doi: 10.1021/acs.langmuir.4c02753 (PMC11526376; doi:10.1021/acs.langmuir.4c02753)
Supplement: Supplementary file 1 — la4c02753_si_001.pdf [file la4c02753_si_001.pdf]

Supporting Information

Supraparticles from Cubic Iron Oxide Nanoparticles: Synthesis, Polymer Encapsulation, Functionalization, and Magnetic Properties

Lea R. Klauke[1], Michael Kampferbeck[1], Malte Holzapfel[2], Neus Feliu[2], Benedikt Sochor[3], Sarathlal Koyiloth Vayalil[3,4], Andreas Meyer[1], Tobias Vossmeyer[1,]*

[1]Institute of Physical Chemistry, University of Hamburg, Grindelallee 117, 20146 Hamburg, Germany

[2]Center for Applied Nanotechnology (CAN), Fraunhofer Institute for Applied Polymer Research (IAP), Grindelallee 117, 20146 Hamburg, Germany

[3]Deutsches Elektron Synchrotron (DESY), Notkestraße 85, 20607 Hamburg, Germany

[4]Applied Science Cluster, University of Petroleum and Energy Studies (UPES), Dehradun 248007, India

Table of contents

SI 1 – Syntheses of differently sized and shaped iron oxide nanocrystals and their characterization 2

SI 2 – Synthesis of 4-Vinylbenzyl azide 7

SI 3 – Calculation of the BiB-UDPA amount for a ligand exchange reaction 7

SI 4 – Mechanism of AGET ATRP…………….……………………………………….……...9

SI 5 – Synthesis of supraparticles and ligand exchange 10

SI 6 – Encapsulation and surface functionalization of supraparticles 14

SI 7 – Arrangement of nanocrystals inside the supraparticles 17

SI 8 – Superparamagnetic properties of supraparticles..20

SI 1 – Syntheses of differently sized and shaped iron oxide nanocrystals and their characterization

The synthesis of spherical superparamagnetic iron oxide nanoparticles (SPIONs) was conducted following the protocol of Yu et al.[1] The reactants were α-FeOOH (2.08 g, 23.4 mmol, goethite), oleic acid (OA, 46.8 mL, 148 mmol), and 1-octadecene (ODE, 100 mL). Size and size distributions of obtained SPIONs were characterized using transmission electron microscopy (TEM) and dynamic light scattering (DLS). The organic fraction was determined via thermogravimetric analysis (TGA). The results of the mentioned characterization techniques can be found in Figure S1.

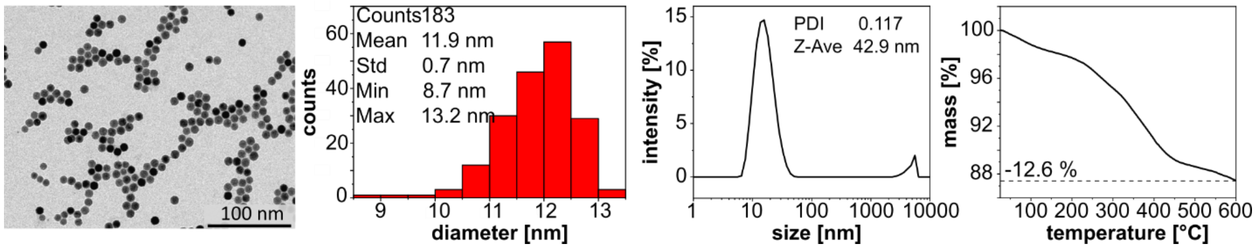

Figure S1: TEM image and histogram, DLS intensity distribution and TGA curve of 12 nm sized spherical SPIONs.

Cubic SPIONs were synthesized according to Kampferbeck et al.[2] In brief, α-FeOOH and sodium oleate (NaOL) were dispersed in ODE and different amounts of OA. The precise masses of reactants can be found in Table S1. The mixture was vigorously stirred and heated to 320-335 °C under a nitrogen atmosphere (caution: Use of pressure relief valve is required due to repeated boiling delay above 250°C of condensed aqueous H_2O and decomposition products). Nucleation of nanoparticles was observed by a color change from dark brown to black between 100 and 205 minutes (Table S1) after the start of heating. After nucleation, the dispersion was stirred for further 30 minutes at the respective temperature (Table S1), and subsequently cooled to room temperature. Below 170 °C, fresh OA (2.0 mL) was added and at 110 °C the dispersion was diluted with toluene (1:1, v/v). The nanocubes were purified by precipitation with acetone (1:1, v/v) and centrifugation at 8000 g for 8 minutes. The supernatant was removed, and the nanoparticle pellet was redispersed in toluene. This step was repeated with a mixture of methanol/acetone (3:1, v/v) until the dispersion stopped foaming. After centrifugation, few drops of OA were added to the pellet to avoid particle destabilization. After that, the particle dispersion was once again precipitated with acetone (1:1, v/v). The size distribution and shape of SPIONs were characterized via TEM and DLS. The organic fraction was determined by TGA. Results are shown in Figure S2.

Table S1: Synthesis parameters for differently sized iron oxide nanocubes.

Size [nm]	FeOOH [g (mmol)]	OA [g (mmol)]	NaOL [g (mmol)]	ODE [mL]	Temperature [°C]	Nucleation time [Min]
12.2±1.4	2.00 (22.5)	19.1 (67.7)	6.90 (22.7)	75.0	322	115
14.3±1.2	3.00 (33.7)	28.6 (101)	10.3 (33.9)	75.0	305	230
15.1±1.5	3.00 (33.7)	28.6 (101)	10.3 (33.9)	75.0	325	100
17.6±1.6	3.00 (33.7)	38.2 (135)	10.3 (33.9)	75.0	325	115
23.5±2.4	3.00 (33.7)	38.5 (137)	10.3 (33.9)	75.0	325	-
30.4±2.1	3.00 (33.7)	57.7 (205)	10.3 (33.9)	75.0	335	205

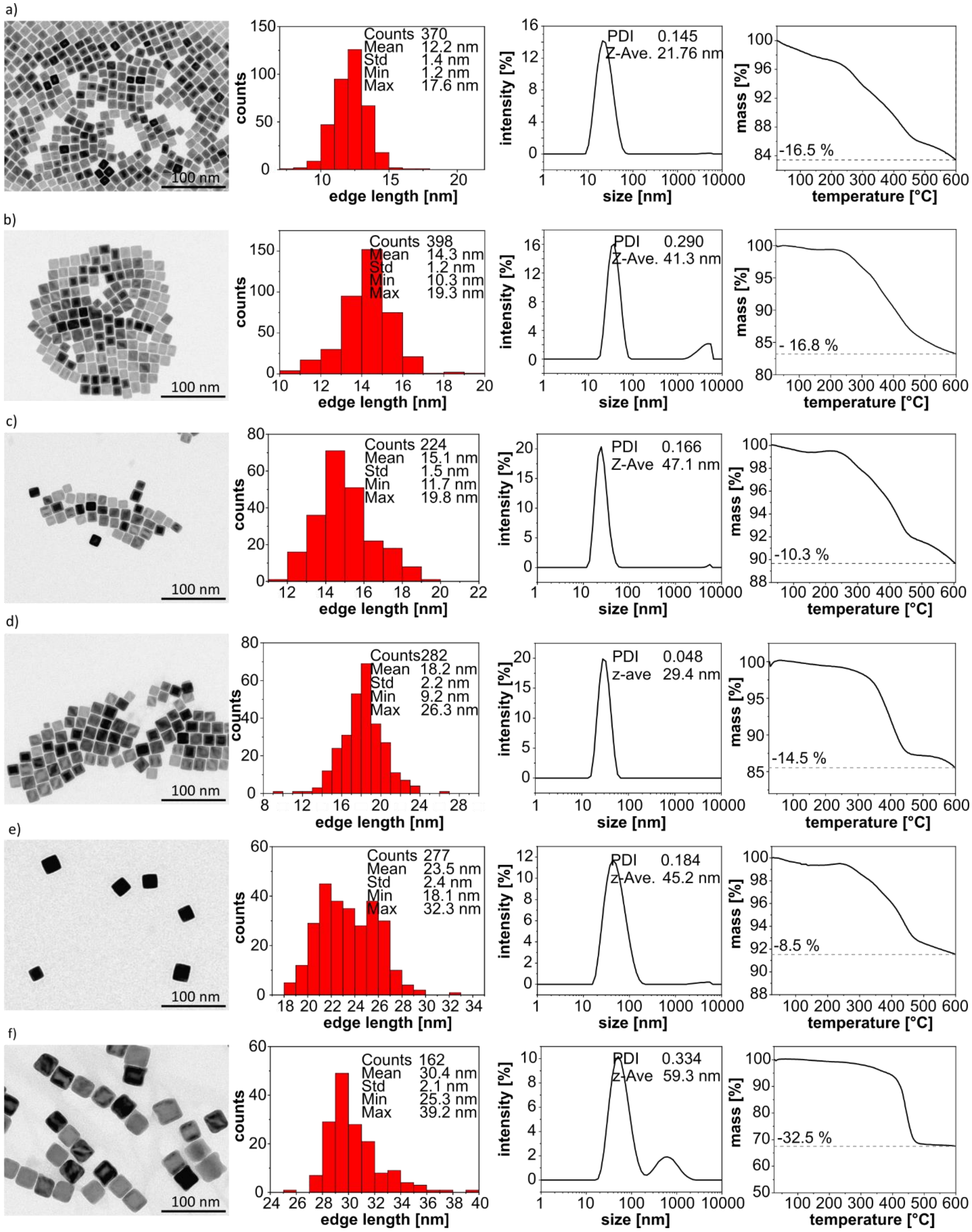

Figure S2: TEM images and histograms, DLS intensity distributions and TGA curves for cubic SPIONs with edge lengths of 12 (a), 14 (b), 15 (c), 18 (d), 24 (e), and 30 nm (f). Note: DLS distributions show the hydrodynamic diameter, including the ligands and solvent shell.

TEM images of 12, 14, 15, 18, and 24 nm sized nanoparticles reveal well-defined shapes and small standard deviations <12% of the nanocrystal edge length or diameter. However, the TEM image of the 30 nm sized nanocubes shows some nanocrystals with rounded corners, similar as reported previously.[3]

Most DLS measurements show monomodal size distributions with polydispersity indices (PDIs) usually below 0.2, indicating narrow size distributions. Therefore, DLS measurements are in good agreement with TEM observations (note: DLS size distributions show the hydrodynamic radius, including the ligands and solvent shell surrounding the nanoparticles). The DLS size distribution of the 14 and 30 nm sized nanocubes additionally shows a low intensity peak at around 5000 nm or 600 nm, which indicates aggregation of some nanocrystals. However, in intensity-weighted size distributions, the number fraction of larger particles is overestimated since the scattering intensity scales with the diameter to the power of 6. Therefore, we assume that only a small fraction of the nanocubes is aggregated. This aggregation was presumably caused by the repeated purification steps using methanol, which can cause destabilization of the nanoparticles due to ligand stripping.

TGA measurements indicate pronounced mass losses between about 200 °C to 600 °C due to decomposition of the OA ligand. TGA mass losses of ~12 nm sized spherical SPIONs are lower compared to cubic SPIONs of the same size. The corresponding grafting densities (GDs, calculated according to ref.[4]) are 2.7 ligands/nm² and 4.5 ligands/nm², respectively. The reference value of the GD corresponding to a monolayer coverage of OA on magnetite nanoparticle surfaces is between 2.8 and 4.2 ligands/nm².[5,6] The GDs of the cubic SPION samples with sizes of 14, 15, 18 and 24 nm are 5.3, 3.2, 5.7, and 4.1 ligands/nm². For 30 nm sized cubes, the GD is determined to be 26.7 ligands/nm² and, hence, is roughly one order of magnitude higher than expected for a monolayer coverage. We assume that the increased size of the nanocubes preferentially causes

aggregation and thus, a larger excess of ligand is required to keep the SPIONs colloidally stabilized.

SI 2 – Synthesis of 4-Vinylbenzyl azide

The synthesis of 4-vinylbenzyl azide was conducted following the approach of Albuszis et al.[7] Briefly, in a roundbottom flask, sodium azide (9.2 g, 0.14 mol, 2.0 eq) was dissolved in *N,N*-Dimethylformamide (DMF) (70 mL) under nitrogen atmosphere. Subsequently, 4-vinylbenzyl chloride (10 mL, 71 mmol, 1.0 eq) was added and the reaction mixture changed from pale yellow solution to an orange suspension. The reaction mixture was stirred under inert N_2-atmosphere at room temperature for 16 h and then quenched with brine (100 mL). The bright yellow solution was extracted three times with diethyl ether (100 mL). The combined organic phases were dried over sodium sulfate, filtered, and the solvent was removed by distillation under reduced pressure. The product was a yellow liquid (11 g, 69 mmol, 97%).

^{1}H NMR (400 MHz, $CDCl_3$, δ): 7.46 – 7.28 (m, 4H, *H*-Ar), 6.77 (dd, *J* = 17.6, 10.9 Hz, 1H, C*H*=CH_2), 5.82 (d, *J* = 17.6 Hz, 1H, Ar-CH-CH_2), 5.33 (d, *J* = 10.9 Hz, 1H, Ar-CH-CH_2), 4.32 (s, 2H, CH_2). Impurities: DMF, diethyl ether.

SI 3 – Calculation of the BiB-UDPA amount for a ligand exchange reaction

The amount of 11-(2-bromoisobutyrate)-undecyl-1-phosphonic acid (BiB-UDPA) used to exchange initial OA ligand on the SPION surface was calculated using the following equations based on the previous work of Kampferbeck et al.[4] First, the surface area A_C of one SP was

calculated from the DLS hydrodynamic radius r using Equation S1. The number of SPs N in the sample was estimated under the approximating assumption that the SPs have a density ρ of ρ=5.2·10^{-18} mg/nm^3 (corresponding to the bulk density of magnetite)[8] and by using the hydrodynamic radius r and the total sample weight m, as shown in Equation S2.

$$A_C = 4\,\pi\, r^2 \tag{S1}$$

$$N = \frac{3m}{4\pi \cdot \rho \cdot r^3} \tag{S2}$$

Multiplication of the number of SPs (S2) with the surface area of one SP (S1) returned the overall surface area A_{sample} shown in Equation S3.

$$A_{sample} = A_C\, N \tag{S3}$$

Finally, the mass of BiB-UDPA $m_{BiB\text{-}UDPA}$ was calculated using Equation S4 based on the Avogadro constant N_A, the molar mass M of BiB-UDPA, the surface area of the sample A_{sample} (in nm^2) and using a grafting density of 3.3 ligands/nm^2 on the SPIONs.[4,9] Equation S5 summarizes all calculations in one equation.

$$m_{BiB\text{-}UDPA} = \frac{3.3\ \text{nm}^{-2} \cdot A_{sample} \cdot M}{N_A} \tag{S4}$$

$$m_{BiB\text{-}UDPA} = \frac{9.9\ \text{nm}^{-2}\ m \cdot M}{\rho \cdot r \cdot \text{N}_A} \tag{S5}$$

SI 4 – Mechanism of AGET ATRP

Figure S3a illustrates the proposed mechanism of the activators generated by electron transfer (AGET) atom transfer radical polymerization (ATRP). The initiator ligand BiB-UDPA is grafted to the SPION surface inside the SPs. The Cu^+ catalyst is formed via *in situ* reduction of Cu^{2+}/BPMODA (*N,N*-bis(2-pyridylmethyl)octadecylamine) using ascorbic acid (AsAc) and initiates the polymerization. The polymer chain growth proceeds via the consumption of the added styrene-derived monomers. Further, every growing polymer chain is in an equilibrium with a dormant species. Activation/deactivation occurs via redox-reaction of the copper-based catalyst. A detailed discussion of the mechanism can also be found in refs.[10–12]

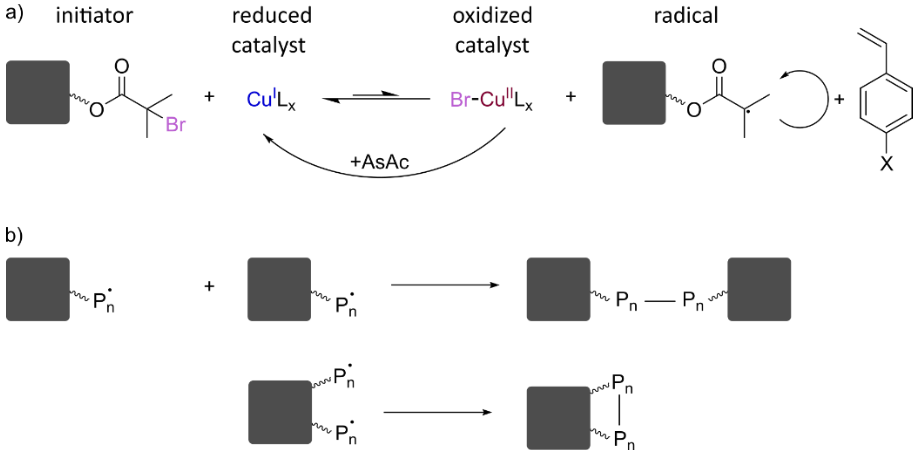

Figure S3. a) Proposed mechanism of the AGET ATRP on the SP surface. b) Possible self-termination reactions during AGET ATRP.

After the initiation, the chain growth continues until the monomer is consumed or irreversible termination reactions, e.g., self-recombination (cf. Figure S3b) occur. Such self-recombination reactions happen likely between polymer chains grafted in close distance to each other, broadening the final polymer's molecular weight distribution[13]. Further, every polymerization in this work was conducted using 50 % divinylbenzene (DVB) of the total monomer volume. DVB acts as

crosslinker of the polymer chains, without self-recombination. However, self-recombination reactions between neighboring polymer chains (cf. Figure S3b) would increase the crosslinking density and terminate the polymerization of the respective chain irreversibly.

As mentioned in the main document, there are mainly three controlled radical polymerization techniques, i.e., ATRP[14,15], reversible addition-fragmentation chain transfer (RAFT)[16] polymerization, and nitroxide-mediated polymerization (NMP)[17]. However, the corresponding mechanisms are different. ATRP relies on the reversible activation and deactivation of active radicals via reversible redox reactions of metal-based catalysts (cf. Figure S3).[10,13] In contrast, RAFT polymerizations use thiocarbonylthio or thiocarbonylsulfanyl compounds to enable chain transfer reactions. In this way, switching between active and dormant species becomes possible.[13] NMP also shows an equilibrium between radical and dormant species, which is based on the activation/deactivation via control agents, e.g. nitroxides.[18]

SI 5 – Synthesis of supraparticles and ligand exchange

During the optimization of the evaporation induced self-assembly (EISA) process, we observed that the way of mixing the nanoparticles with the surfactant solution influenced the size distribution of resulting clusters. Combining the aqueous dodecyltrimethylammonium bromide (DTAB) solution (1.0 mL, 20 mg/mL) with the SPION solution (edge length: 15.1±1.5 nm, concentration: 10 mg/mL in chloroform) without simultaneous vortexing resulted in two clearly separated phases. Subsequently vortexing this two-phase system provided nanoparticle clusters of different sizes after solvent evaporation, as indicated by the bimodal distribution shown in Figure S4a (blue line, PDI: ~ 0.5). Additionally, magnetic separation of the prepared clusters was

incomplete when applying a 1.5 T magnet. A dark brown supernatant indicated the presence of isolated nanocubes or small clusters that could not be separated.

In contrast, as indicated by the DLS data (Figure S4a, black solid line), the addition of the SPION solution via syringe injection into the DTAB solution under simultaneous vortexing (referred to as "syringe injection method" in the main document) led to the formation of nanoparticle clusters with a monomodal size distribution (PDI: ~0.16). These clusters showed a nearly unchanged size distribution before and after magnetic separation (Figure S4a, black dashed line). We attribute the improved size distribution to the homogeneous size of oil droplets that are formed during the syringe injection procedure, acting as templates for the formation of clustered nanoparticles.

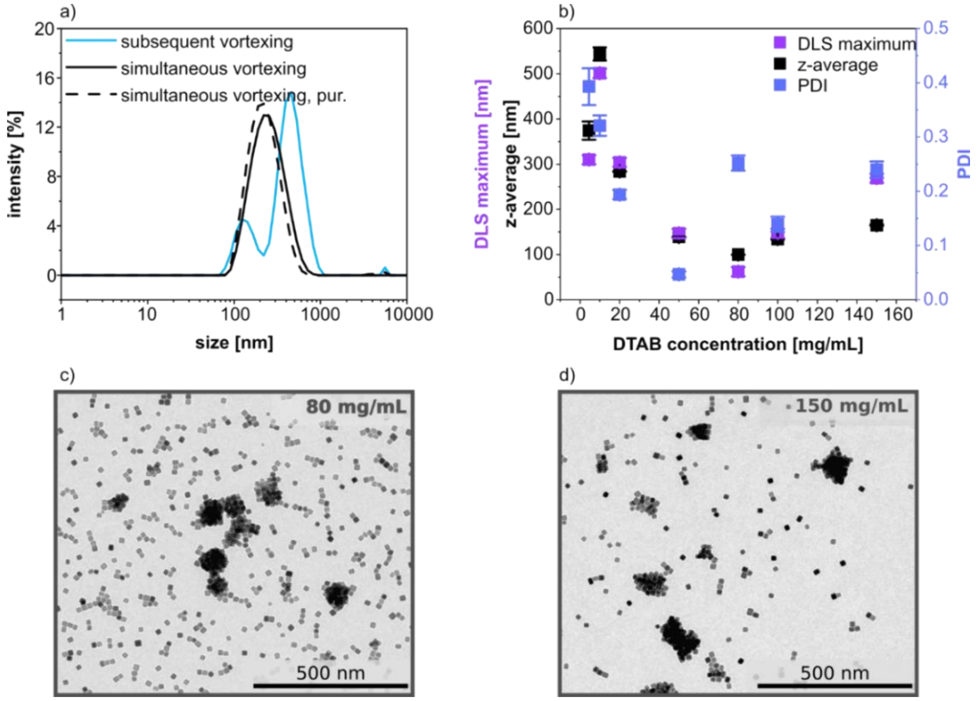

Figure S4. a) DLS intensity distributions of clustered iron oxide nanocubes after solvent evaporation. The nanocube clusters were obtained by either combining the dispersions of nanocubes and DTAB and subsequently vortexing the mixture (blue line) or by injecting the nanocube dispersion into the DTAB solution under simultaneous vortexing (black solid line). The dashed black line shows the DLS distribution after magnetic purification. b) Dependency of the DLS z-average (black squares), the position of the maximum obtained from the DLS intensity distribution (purple squares) and the PDI (blue squares) on the DTAB concentration. c, d) TEM images of clusters prepared with 80 and 150 mg/mL DTAB, as indicated.

To study the formation of SPs as a function of surfactant concentration, the DTAB concentration was varied between 4.5 and 150 mg/mL. As shown in Figure S4b, the z-average values (black squares) obtained from DLS measurements directly after solvent evaporation increased with increasing DTAB concentration from 4.5 to 10 mg/mL. We attribute this observation to the low concentration of the surfactant, which was close to the critical micelle concentration (cmc; reported values: 4.3 mg/mL[19] and 4.5 mg/mL[20]). Hence, the DTAB concentration of 4.5 mg/mL was most likely not sufficient for the stabilization of SPs. Further increasing the DTAB concentration from 10 to 80 mg/mL resulted in decreasing z-average values from ~550 nm to ~100 nm. The TEM images shown in Figure 2b (main document) confirm the formation of spherical SPs with diameters of ~200 to ~400 nm at the DTAB concentration of 20 mg/mL. In contrast, SPs of defined shape were not observed at the DTAB concentration of 80 mg/mL (cf. Figure S4c). These findings are attributed to the stabilization of smaller oil droplets at higher surfactant concentrations. Since smaller droplets contain less nanoparticles, smaller particle clusters are formed during solvent evaporation. Even further increasing the DTAB concentration to 150 mg/mL resulted in a moderate increase of the z-average size from ~100 nm to ~160 nm. However, TEM images of samples prepared with a DTAB concentration of 150 mg/mL did not indicate the formation of SPs with defined shape (cf. Figure S4d). Previously, Paquet et al.[21] observed similar trends for the preparation of SPs via the EISA process using sodium dodecyl sulfate (SDS) as surfactant in an octanol/water emulsion. They explained the initial increase of the cluster sizes at SDS concentrations slightly above the cmc with the formation of aligned SDS molecules at high shear forces. Such alignment causes fracture of the viscous flow of the continuous phase and, thus, decreases the effective shear force, resulting in increased oil droplet sizes.

In general, the PDI values (blue squares) of the SPs and clustered nanoparticles produced at different DTAB concentrations reveal similar trends as the z-averages (Figure S4b). First, the PDI decreased from roughly 0.4 at a DTAB concentrations of 4.5 mg/mL to below 0.1 at 50 mg/mL. Small PDIs of ~0.1 indicate narrow size distributions of the formed nanoparticle assemblies. Further increasing the DTAB concentration led to somewhat increased PDI values between 0.1 and 0.3.

The size distribution of SPs and clustered nanoparticles can also be evaluated by comparing the z-average value to the position of the maximum of the intensity distributions.[22] The dependency of the latter on the DTAB concentrations can also be found in Figure S4b (violet squares). The overall trend of decreasing sizes (indicated by the intensity distributions) with increasing DTAB concentrations agrees with the trend of the z-average values (black squares). At the DTAB concentration of 20 and 50 mg/mL the maximum of the intensity distribution closely matches the z-average value, indicating a monomodal size distribution. Based on the findings presented above and the increased magnetophoretic mobility of larger SPs, we conducted the fabrication of spherical SPs for subsequent experiments using a DTAB concentration of 20 mg/mL.

To proof the exchange of the initial oleic acid ligands on the nanocubes within the SPs for the ATRP initiator ligand BiB-UDPA, we treated SP samples with a BiB-UDPA amount corresponding roughly to a monolayer coverage of the SP surface (cf. Chapter SI 3) and a 30-fold higher BIB-UDPA amount. Figure S5 shows photographs of respective samples with SP concentrations of ~10 mg/mL. In the absence of external magnetic fields (a), the dispersions remained stable. However, after the samples were placed close to two disk magnets (NdFeB, N42, holding power ~3.7 kg) the SPs mixed with a concentration corresponding to a monolayer of BiB-

UDPA (1x) could be collected magnetically much more efficiently than in the case of the sample mixed with the 30-fold higher concentration of BIB-UDPA (30x, Figure S5b).

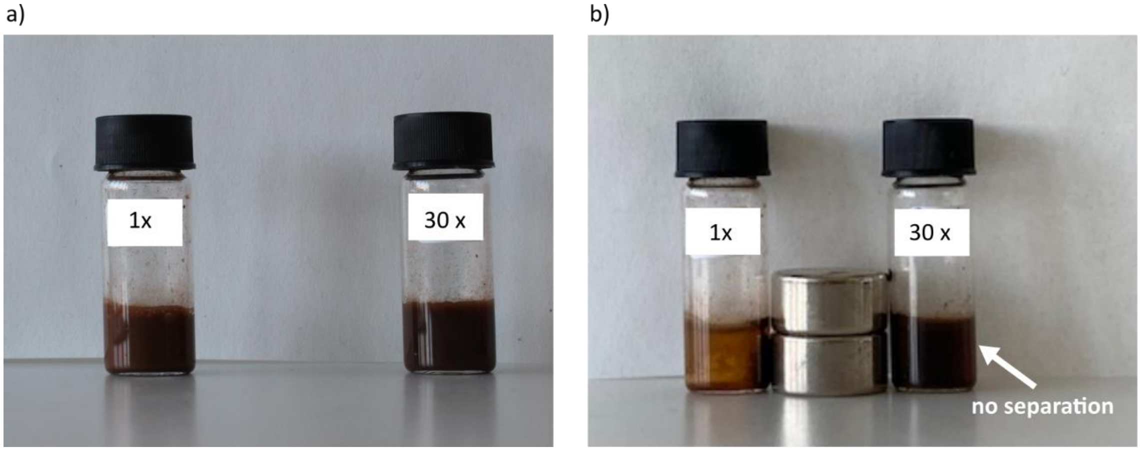

Figure S5. SP dispersions treated with either an amount of BIB-UDPA corresponding to a monolayer coverage of the SPs (1x) or with a 30-fold higher amount (30x) in the absence (a) and presence (b) of an external magnetic field (two NdFeB disk magnet, N42, holding power ~3.7 kg).

SI 6 – Encapsulation and surface functionalization of supraparticles

To adjust the polymer shell thickness, we increased the amount of monomer (styrene/DVB) used for the ATRP reaction stepwise from 70 to 280 µL. The TEM image in Figure S6a presents the thin polymer shell of SPs encapsulated with 70 µL monomer. Further, TGA data of all encapsulated SPs confirm an increasing polymer shell thickness with increased monomer volumes (Figure S6b). Before TGA measurements, the samples were magnetically separated to remove excess DTAB and dried. Moreover, Figure S7 shows TEM images of as assembled SPs and SPs after the encapsulation. The samples were stored for 6 months (as assembled SPs) or 33 months (encapsulated SPs) in the dispersions before conducting the TEM measurements. All SPs prepared in these experiments are based on cubic SPIONs with an edge length of 15.1±1.5 nm.

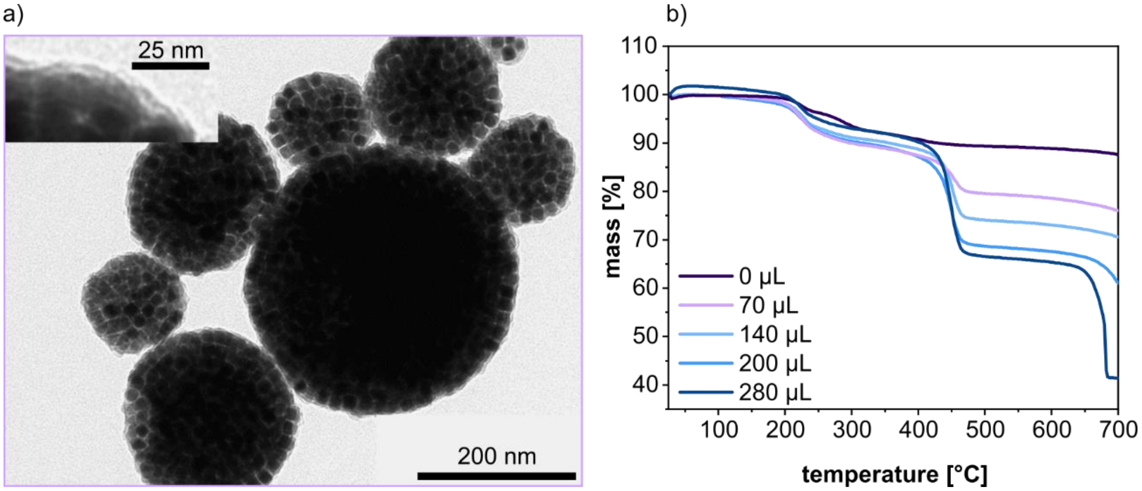

Figure S6. a) TEM image of SPs encapsulated with 70 µL monomer (styrene/DVB) indicate the formation of a thin polymer shell. b) TGA data confirm increasing polymer shell thicknesses with increasing amount of added monomer.

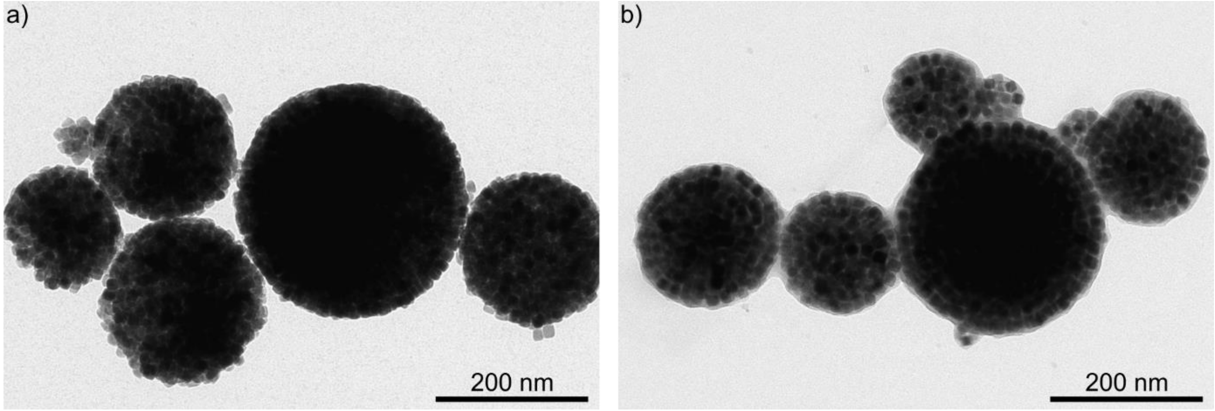

Figure S7. TEM image of as assembled SPs (a) and SPs after encapsulation (b). The samples were stored for 6 or 33 months in dispersion before the TEM measurements, respectively.

To obtain insights into the size distribution of the SPs and their stability against aggregation, we characterized the SPs before and after the encapsulation using DLS. The DLS intensity distributions of the SPs prior and after the encapsulation can be seen in Figure S8. Additionally, the corresponding PDIs and z-average values (z-ave.s) are listed in Table S2 for 70-280 µL added monomer. In general, the distributions are shifted to higher sizes after the encapsulation (red

curves). This shift cannot only be explained by the presence of the polymer shell. Probably, the SPs aggregated to some extent during the encapsulation and purification procedures. Z-average values listed in Table S2 support this assumption. Further, for SPs encapsulated with 70 µL and 140 µL of monomer, DLS intensity distributions were measured directly after the ATRP and again after the purification via magnetic separation. These measurements suggest that aggregation occurred mainly during the magnetic purification step as the z-average values increased more drastically after the purification than after the ATRP.

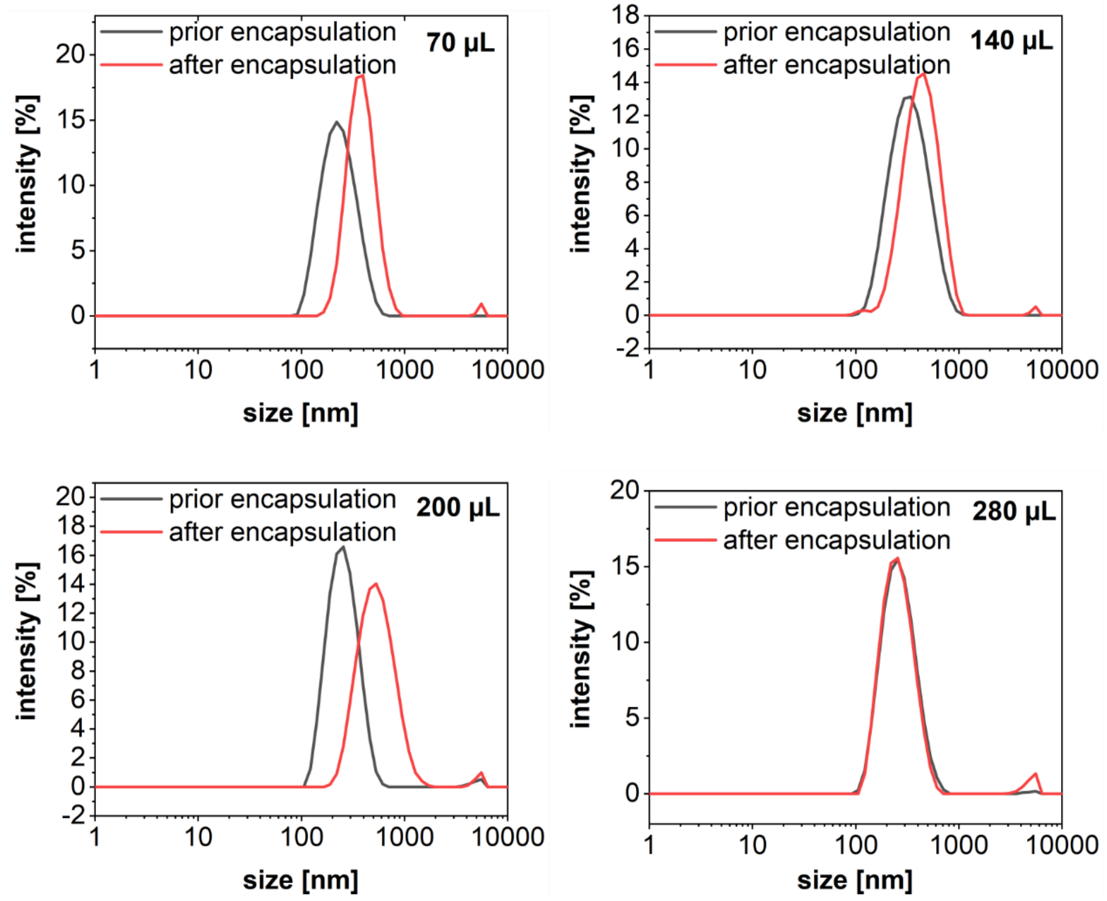

Figure S8. DLS size distributions prior (black curves) and after the encapsulation (red curves) of SPs using different monomer volumes, ranging between 70-280 µL.

Table S2: PDI and z-average values from DLS measurements prior and after ATRP encapsulation, as well as after magnetic purification (pur.), of SPs encapsulated using monomer volumes between 70-280 μL.

	**70 μL**			**140 μL**			**200 μL**		**280 μL**	
	Prior ATRP	After ATRP	After pur.	Prior ATRP	After ATRP	After pur.	Prior ATRP	After pur.	Prior ATRP	After pur.
PDI	0.117	0.196	0.402	0.138	0.171	0.267	0.213	0.235	0.162	0.203
Z-ave. [nm]	214.4	260.5	481.5	301.7	278.0	446.3	256.4	536.3	251.2	256.1

SI 7 – Arrangement of nanocrystals inside the supraparticles

Small-angle X-ray scattering (SAXS) data of SPs based on spherical and cubic nanocrystals (~12 nm) before and after the encapsulation with styrene and DVB are shown in Figure S9. The parameters of the fit function (lattice constant, nearest neighbor distance, SPION radius, domain size) for a face centered cubic (fcc) lattice of the spherical SPIONs are listed in Table S3. For the SPs from cubic SPIONs, the scattering vector q was determined from SAXS curves as indicated in Figure S9c, d and Figure S10. Yellow highlighted areas indicate the range of scattering vectors, in which the peak analysis was conducted. The data shown in Figure S9 were obtained from dried SPs on Kapton foil. SAXS data shown in Figure S10 were measured using SPs dispersed in aqueous DTAB solution (at PETRA III, DESY).

Equation S6 was used to calculate the lattice parameter L from the scattering vector q of the first peak´s position in the SAXS curve. For SPs assembled from cubic SPIONs we assumed a simple cubic lattice for the SPION arrangement inside the supraparticles (SPs).

$$L = \frac{2\pi}{q} \tag{S6}$$

The edge-to-edge distances were estimated by subtracting the SPION edge lengths *s* (determined by transmission electron microscopy, TEM). The resulting data are presented in Table S4**.**

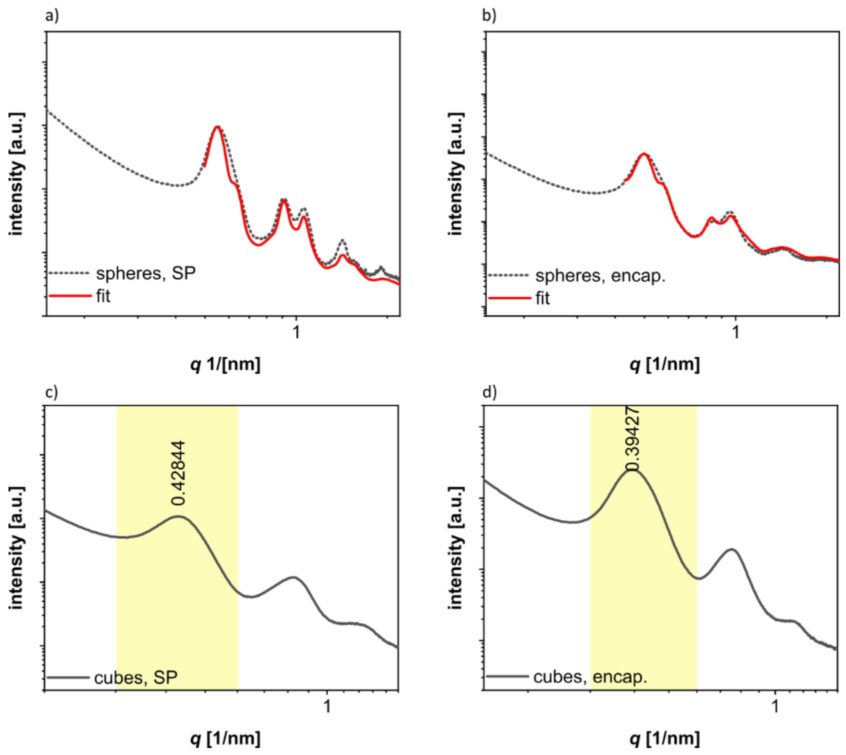

Figure S9: a, b) SAXS curves (dotted lines) with curve fits (solid lines) of SPs from ~12 nm sized spherical nanocrystals before (a) and after (b) the encapsulation using 70 µL monomer (styrene/DVB). c, d) SAXS curves of SPs from ~12 nm cubic nanocrystals before (c) and after encapsulation (d) using 70 µL monomer (styrene/DVB). The yellow highlighted areas indicate the *q* vector range, which was used for the peak analysis. For these measurements the SPs were deposited onto Kapton foil.

Table S3: Lattice constants (fcc), nearest neighbor distances, SPION radii, and domain sizes of SPs from spherical SPIONs prior (SP) and after the encapsulation (encap.) extracted from SAXS data. For these measurements, the samples were deposited onto Kapton foil.

Sample	fcc[nm]	Nearest neighbor [nm]	Radius [nm]	Domain [nm]
Spherical, SP	19.5	13.8	6.3	106
Spherical, encap.	21.5	15.2	6.4	109

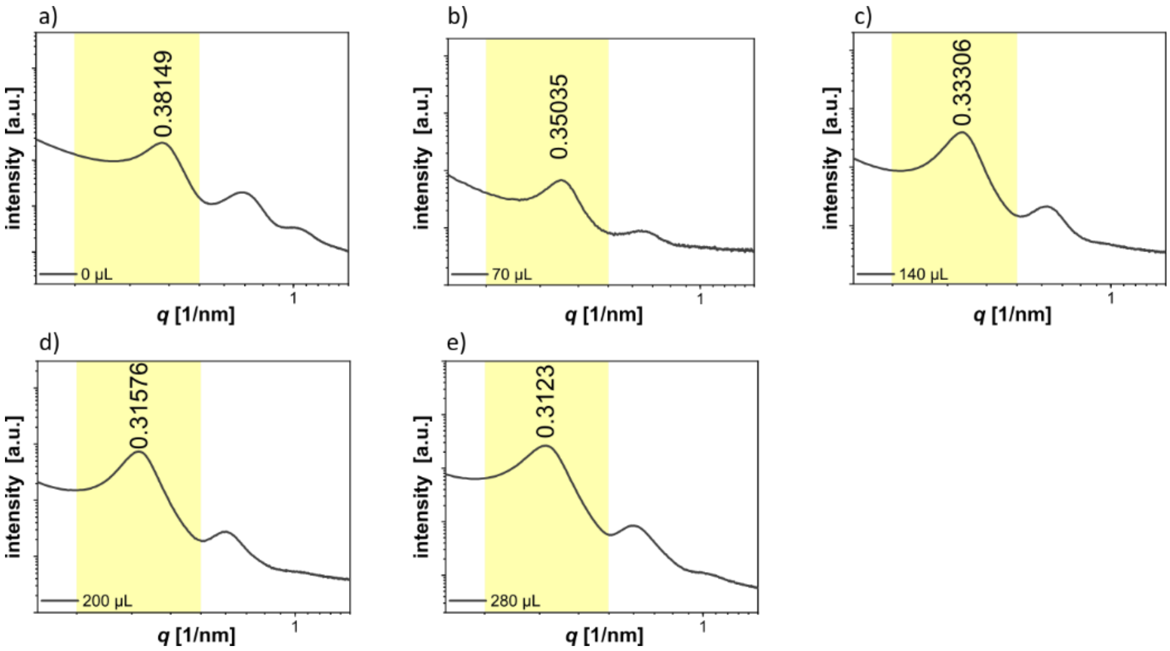

Figure S10: SAXS curves of SPs based on cubic SPIONs before (a) and after encapsulation using different volumes of monomer: 70 µL (b), 140 µL (c), 200 µL (d), and 280 µL (e). The scattering vector was determined via peak analysis of data limited to the *q* vector range highlighted in yellow. These measurements were conducted using SPs dispersed in DTAB solution (at PETRA III, DESY).

Table S4: Scattering vectors *q* obtained from SAXS curves, calculated lattice parameters *L*, and estimated edge-to-edge distances obtained by subtracting the edge length *s* (determined by TEM) of the cubic SPIONs from *L*. Data from dispersed SPs (measured at PETRA III, DESY) are highlighted in yellow. Data obtained using SPs dried on Kapton foil are not highlighted.

Monomer volume [µL]	Scattering vector *q* [1/nm]	Lattice parameter *L* [nm]	Edge length *s* [nm]	approximate edge-to-edge distance [nm]
0 (before enc.)	0.428	14.7	12.2±1.4	2.5
70	0.394	15.9	12.2±1.4	3.7
0 (before enc.)	0.381	16.5	15.1±1.5	1.4
70	0.350	18.0	15.1±1.5	2.9
140	0.333	19.0	15.1±1.5	3.9
200	0.316	19.9	15.1±1.5	4.8
280	0.312	20.1	15.1±1.5	5.0

SI 8 – Superparamagnetic properties of supraparticles

We compared the magnetization curves obtained from vibrating sample magnetometer (VSM) measurements of encapsulated individual SPIONs to encapsulated SPs. After normalizing the data to the inorganic mass fraction (i.e., excluding the polymer shell, ligands, residual surfactant) and correcting for the water signal by subtracting a water measurement, the VSM curves of both samples revealed the same magnetic behavior, as shown in Figure S11. The saturation magnetization was ~40 emu/g (based on the mass of inorganic material) in both samples, which is below the magnetization of bulk magnetite of about 84 emu/g.[23] According to earlier studies, we attribute the lower magnetization to surface defects in the nanocrystals[23] and the presence of mixed iron oxide modifications.[24]

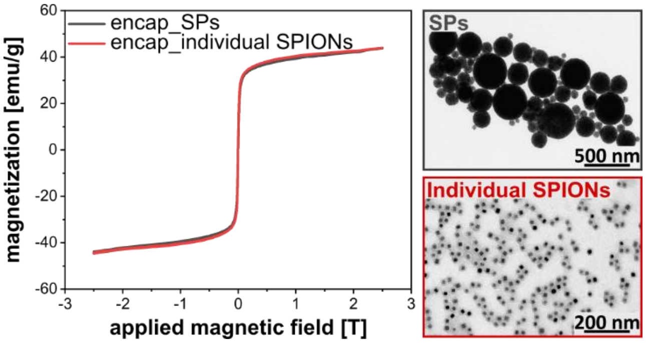

Figure 11. Magnetization curves of individually encapsulated iron oxide nanocubes (15 nm edge length, red curve) and SPs (black curve) from the same nanocubes. Both samples were encapsulated using the same monomer volume (140 µL styrene/DVB). The VSM measurements were conducted with aqueous suspensions of the samples. The data were normalized to the mass of the nanocubes excluding the organic fraction. Panels to the right show TEM images of the encapsulated SPs and nanocubes used in these experiments.

Figure S12a shows VSM curves of SPs (grey curve) and individual nanocubes (red curve), both encapsulated with 140 µL styrene/DVB (1:1, v/v) after correction for the water signal. In both

samples, the nanocubes had an edge length of ~15 nm. The VSM curves normalized to the total mass of the nanocomposite show different magnetizations at saturation of ~27 emu/g for the SPs and ~23 emu/g for the individually encapsulated SPIONs. Such differences are caused by different organic fractions of ~45 % and ~50 % (obtained from TGA, Figure S12b), most likely resulting from different purification methods after encapsulation and different surfactants, e.g., DTAB and Brij® S20. Figure S12c shows the TGA data of pure DTAB under nitrogen. The decomposition takes place between ~200-270 °C, as indicated by the mass loss. The same drop can be observed in the TGA data of encapsulated SPs.

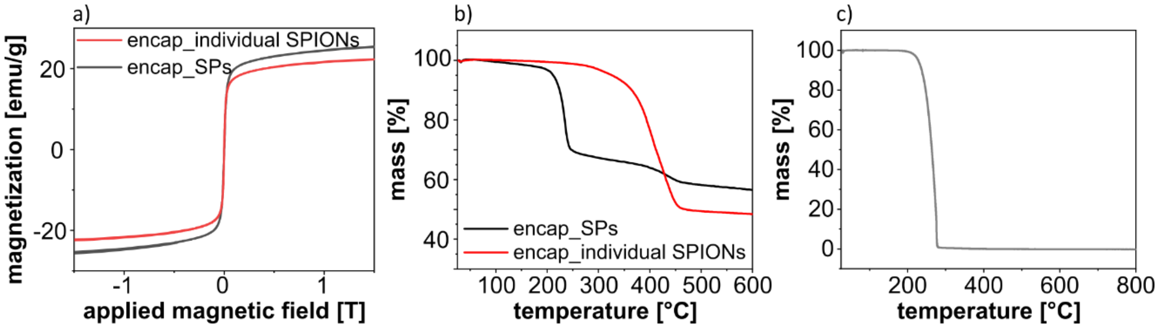

Figure S12: a) VSM curves of SPs (black curve) and individual SPIONs (red curve) encapsulated with 140 µL monomer (styrene/DVB). The curves are normalized to the total mass of the nanocomposite. b) TGA curves of the corresponding samples. c) Reference TGA curve of pure DTAB shows a decomposition between 200-270 °C.

Tuning the magnetization of the encapsulated SPs was achieved by using differently sized SPIONs of 12, 18, 24 and 30 nm edge length. The TEM images in Figure S13 show corresponding SPs based on differently sized SPIONs after the encapsulation with 70 µL styrene/DVB (1:1, v/v), resulting in a thin polymer shell.

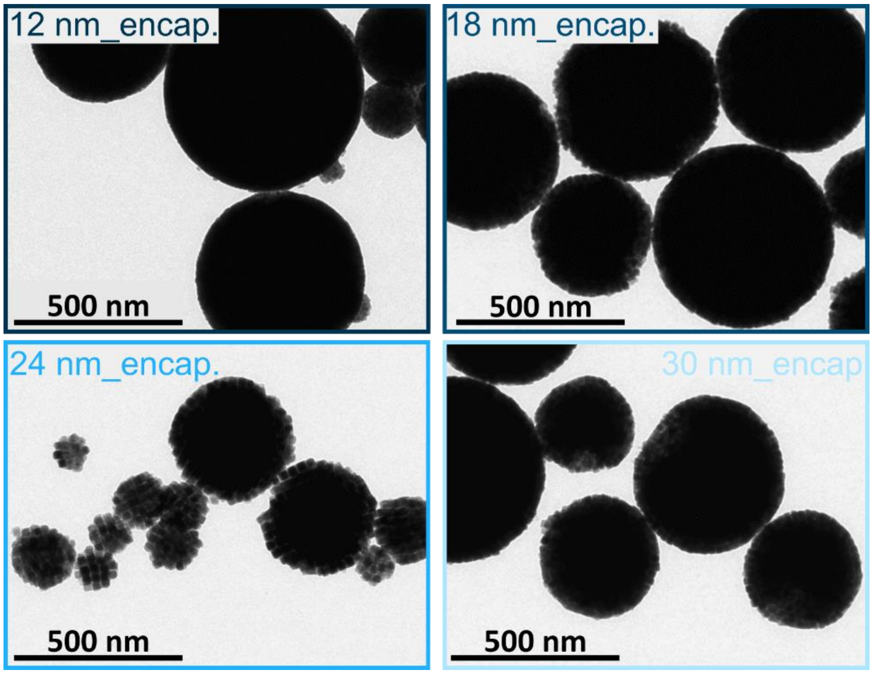

Figure S13: TEM images of SPs based on 12, 18, 24 and 30 nm sized cubic SPIONs encapsulated with 70 µL added monomer (styrene/DVB).

VSM curves of the same samples normalized to the total mass of the SP composites in Figure S14a show the same trend as the VSM curves normalized to the inorganic fraction (Figure 8a, main document). Additionally, TGA curves of the SPs based on the same SPIONs are depicted (Figure S14b). Compared to the TGA curves of SPs in Figure S6b the mass loss of TGA curves in Figure S14b at~200-250 °C is increased. We attribute the increased drop to the decomposition of DTAB (cf. Figure S12c). For VSM analysis, the samples were measured directly in the aqueous dispersion, including the excess surfactant. Hence, for TGA measurements, the samples were not separated magnetically and the excess surfactant was measured as well in order to determine the organic fraction in the VSM measurements. This missing purification step increased the mass loss in TGA measurements at ~200-250 °C compared to the samples in Figure S6b.

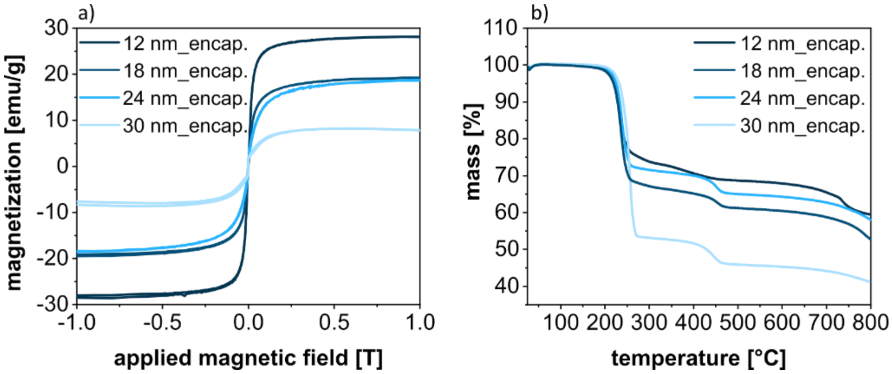

Figure S14: a) VSM curves of SPs from 12 nm (black curve), 18 nm (dark blue curve), 24 nm (medium blue curve) and 30 nm (light blue curve) sized nanocubes normalized to the total mass of the composite material. b) TGA curves of SPs based on the differently sized SPIONs which were used to normalize the VSM data.

References

1. Yu, W. W.; Falkner, J. C.; Yavuz, C. T.; Colvin, V. L. Synthesis of monodisperse iron oxide nanocrystals by thermal decomposition of iron carboxylate salts. *Chem. Commun.* **2004**, 2306–2307.

2. Kampferbeck, M.; Klauke, L. R.; Weller, H.; Vossmeyer, T. Little Adjustments Significantly Simplify the Gram-Scale Synthesis of High-Quality Iron Oxide Nanocubes. *Langmuir* **2021**, *37*, 9851–9857.

3. Feld, A.; Weimer, A.; Kornowski, A.; Winckelmans, N.; Merkl, J. P.; Kloust, H.; Zierold, R.; Schmidtke, C.; Schotten, T.; Riedner, M.; Bals, S.; Weller, H. Chemistry of Shape-Controlled Iron Oxide Nanocrystal Formation. *ACS Nano* **2019**, *13*, 152–162.

4. Kampferbeck, M.; Vossmeyer, T.; Weller, H. Cross-Linked Polystyrene Shells Grown on Iron Oxide Nanoparticles via Surface-Grafted AGET-ATRP in Microemulsion. *Langmuir* **2019**, *35*, 8790–8798.

5. Roonasi, P.; Holmgren, A. A Fourier transform infrared (FTIR) and thermogravimetric analysis (TGA) study of oleate adsorbed on magnetite nano-particle surface. *Appl. Surf. Sci.* **2009**, *255*, 5891-5895.

6. Zhang, L.; He, R.; Gu, H.-C. Oleic acid coating on the monodisperse magnetite nanoparticles. *Appl. Surf. Sci.* **2006**, *253*, 2611-2617.

7. Albuszis, M.; Roth, P. J.; Exnowitz, F.; Wong, D. L.; Pauer, W.; Moritz, H.-U. Synthesis and in-depth characterization of reactive, uniform, crosslinked microparticles based on free radical copolymerization of 4-vinylbenzylazide. *Polym. Chem.* **2016**, *7*, 1168-1180.

8. Knipping, J. L.; Webster, J. D.; Simon, A. C.; Holtz, F. Accumulation of magnetite by flotation on bubbles during decompression of silicate magma. *Sci. Rep.* **2019**, *9*, 3852-3858.

9. Daou, T. J.; Begin-Colin, S.; Grenéche, J. M.; Thomas, F.; Derory, A.; Bernhardt, P.; Legaré, P.; Pourry, G. Phosphate adsorption properties of magnetite-based nanoparticles. *Chem. Mater.* **2007**, *19*, 4494-4505.

10. Min, K.; Matyjaszewski, K. Atom transfer radical polymerization in aqueous dispersed media. *Cent. Eur. J. Chem.* **2009**, *7*, 657–674.

11. Min, K.; Gao, H.; Matyjaszewski, K. Preparation of Homopolymers and Block Copolymers in Miniemulsion by ATRP Using Activators Generated by Electron Transfer (AGET). *J. Am. Chem. Soc.* **2005**, *127*, 3825–3830.

12. Jakubowski, W.; Matyjaszewski, K. Activator Generated by Electron Transfer for Atom Transfer Radical Polymerization. *Macromolecules* **2005**, *38*, 4139–4146.

13. Truong, N. P.; Jones, G. R.; Bradford, K. G. E.; Konkolewicz, D.; Anastasaki, A. A comparison of RAFT and ATRP methods for controlled radical polymerization. *Nat. Rev. Chem.* **2021**, *5*, 859–869.

14. Kato, M.; Kamigaito, M.; Sawamoto, M.; Higashimura, T. Polymerization of Methyl Methacrylate with the Carbon Tetrachloride/Dichlorotris(triphenylphosphine)ruthenium(II)/-Methylaluminium Bis(2,6-di-*tert*-butylphenoxide) Initiiating System: Possilbility of Living Radical Polymerization. *Macromolecules* **1995**, *28*, 1721–1723.

15. Wang, J.-S.; Matyjaszewski, K. Controlled /"Living" Radical Polymerization . Atom Transfer Radical Polymerization in the Presence of Transition-Metal Complexes. *J. Am. Chem. Soc.* **1995**, *117*, 5614–5615.

16. Chiefari, J.; Chong, Y. K.; Ercole, F.; Krstina, J.; Jeffery, J.; Le, T. P. T.; Mayadunne, R. T. A.; Meijs, G. F.; Moad, C. L.; Moad, G.; Rizzardo, E.; Thang, S. H. Living Free-Radical Polymerization by Reversible Addition-Fragmentation Chain Transfer: The RAFT Process. *Macromolecules* **1998**, *31*, 5559–5562.

17. Grubbs, R. B. Nitroxide-Mediated Radical Polymerization: Limitations and Versatility. *Polym. Rev.* **2011**, *51*, 104–137.

18. Nicolas, J.; Guillaneuf, Y.; Lefay, C.; Bertin, D.; Gigmes, D.; Charleux, B. Nitroxide-mediated polymerization. *Prog. Polym. Sci.* **2013**, *38*, 63–235.

19. Bahri, M. A.; Hoebeke, M.; Grammenos, A.; Delanaye, L.; Vandewalle, N.; Seret, A. Investigation of SDS, DTAB and CTAB micelle microviscosities by electron spin resonance. *Colloids Surf., A* **2006**, *290*, 206–212.

20. Shah, S. K.; Chatterjee, S. K.; Bhattarai, A. The Effect of Methanol on the Micellar Properties of Dodecyltrimethylammonium Bromide (DTAB) in Aqueous Medium at Different Temperatures. *J. Surfactants Deterg.* **2016**, *19*, 201–207.

21. Paquet, C.; Pagé, L.; Kell, A.; Simard, B. Nanobeads Highly Loaded with Superparamagnetic Nnanoparticles Prepared by Emulsification and Seeded-Emulsion Polymerization. *Langmuir* **2010**, *26*, 5388–5396.

22. Jia, Z.; Li, J.; Gao, L.; Yang, D.; Kanaev, A. Dynamic Light Scattering: A Powerful Tool for In Situ Nanoparticle Sizing. *Colloids Interfaces* **2023**, *7*, 15–32.

23. Kim, T.; Shima, M. Reduced magnetization in magnetic oxide nanoparticles. *J. Appl. Phys.* **2007**, *101*, 516–518.

24. Lak, A.; Kraken, M.; Ludwig, F.; Kornowski, A.; Eberbeck, D.; Sievers, S.; Litterst, F. J.; Weller, H., Schilling, M. Size dependent structural and magnetic properties of FeO-Fe_3O_4 nanoparticles. *Nanoscale* **2013**, *5*, 12286–12295.
